# Unveiling *Pulmonaria rubra* Schott: Phytochemical Characterisation and Evaluation of Its Neuroprotective Potential

**DOI:** 10.3390/ijms27146122

**Published:** 2026-07-08

**Authors:** Ivan Stambolov, Aleksandar Shkondrov, Lyubomira Vusheva, Magdalena Kondeva-Burdina, Ilina Krasteva

**Affiliations:** 1 Department of Pharmacognosy, Faculty of Pharmacy, Medical University of Sofia, 2 Dunav st., 1000 Sofia, Bulgaria; istambolov@pharmfac.mu-sofia.bg (I.S.); 105230@students.mu-sofia.bg (L.V.); ikrasteva@pharmfac.mu-sofia.bg (I.K.); 2 Laboratory of Drug Metabolism and Drug Toxicity, Department of Pharmacology, Pharmacotherapy and Toxicology, Faculty of Pharmacy, Medical University of Sofia, 2 Dunav st., 1000 Sofia, Bulgaria; mkondeva@pharmfac.mu-sofia.bg

**Keywords:** *Pulmonaria rubra*, phytochemicals, phenolics, neuroprotection, antioxidant activity, rosmarinic acid, quantification

## Abstract

*Pulmonaria rubra* (Boraginaceae) is a widely distributed plant in Bulgaria, yet its phytochemical profile and therapeutic potential have remained unexplored. *P. rubra* methanol extract (PRE) was evaluated through phytochemical profiling and in vitro neuroprotective and antioxidant assays. Rat brain synaptosomes, mitochondria and microsomes were treated with PRE alone, and in combination with 6-hydroxydopamine and *tert*-butyl hydroperoxide as toxic agents. The extract exhibited concentration-dependent protective effects in all subcellular models. Additionally, it was tested on *h*MAOA/B and different isoforms of CYP450 enzymes, but it did not show any activity in the tested conditions. In the UHPLC-HRESIMS analysis, 26 secondary metabolites were identified, mainly hydroxycinnamic acids and caffeoyl oligomers, flavonoids, a lignan (globoidnan A), and the terpenoid glycoside roseoside. Seven compounds were identified via UHPLC-UV method using reference compounds: rosmarinic acid, rutin, quercetin-3-*O*-glucoside, astragalin, apigenin-7-*O*-glucoside, apigenin-7-*O*-glucuronide and alcesefoliside, with the latter two being reported for the first time in genus *Pulmonaria*. The quantity of rosmarinic acid in PRE was 4.35%, distinguishing the compound as the main bioactive molecule in the species. Characterized by its high content of rosmarinic acid, *P. rubra* represents a highly viable candidate for subsequent development into standardized phytopharmaceuticals targeting oxidative stress-related neurodegenerative diseases.

## 1. Introduction

Genus *Pulmonaria* (Boraginaceae) comprises 18 flowering species that are widely distributed around Europe and parts of Asia [1]. The plants are known to accumulate various biologically active compounds, such as flavonoids, phenolic acids, alkaloids, and lignans, etc. [2]. For instance, early two-dimensional TLC and HPLC-MS analyses of *P. officinalis* and *P. mollis* established the widespread prevalence of common phenolic compounds, including gallic, chlorogenic, caffeic, and *p*-coumaric acids, alongside flavonoid glycosides such as rutin, hyperoside, and various derivatives of quercetin, apigenin, and luteolin [3,4,5]. More advanced liquid chromatography–tandem mass spectrometry (LC-MS/MS) and NMR investigations of *P. officinalis* have recently mapped a deeper chemical complexity, identifying rare secondary metabolites like yunnaneic acid B, lithospermic acid A, the rare shimobashiric acid C, nitrile glucosides (menisdaurin), and genus-specific lignans such as globoidnans A–B and pulmonariosides A–B [6,7]. Concurrently, quantitative profiles consistently highlight allantoin and the caffeic acid dimer rosmarinic acid as dominant constituents within the genus, while noting the absence of shikonin, a naphthoquinone-type pigment characteristic for Boraginaceae [5,6,8].

This rich and diverse phenolic composition directly underpins the diverse pharmacological profiles observed across *Pulmonaria* species, particularly their robust antioxidant and enzyme-inhibitory capacities. An extract from the leaves of *P. mollis* revealed significant antioxidant activity based on radical-scavenging assay, chelating activity and capacity to protect plasmid DNA against H_2_O_2_ [9]. Yunnaneic acid B, isolated from the aerial parts of *P. officinalis*, showed a significant in vitro antioxidant effect in blood plasma in conditions of peroxynitrile-induced oxidative stress. The cytotoxicity of the compound (≤50 µg/mL) towards peripheral blood mononuclear cells was also examined [7].

The potential in vitro anti-inflammatory effect of extracts from two *Pulmonaria* species, *P. officinalis* and *P. obscura*, was analyzed using COX-2 as a drug target. The activity was measured by ELISA and by evaluation of the enzymatic activity of the peroxidase component of COX with indomethacin as the positive control. It was found that the inhibitory effect of the *P. officinalis* extract was more pronounced [10]. Aqueous and 70% ethanol extracts from the same plant showed high acetylcholinesterase and tyrosinase in vitro inhibitory activity, indicating its potential for treating neurodegenerative diseases [5].

While these natural product scaffolds offer promising therapeutic templates, drug discovery for neurodegenerative disorders remains severely impeded by the complex, multifactorial pathogenesis of these conditions, which simultaneously involve oxidative stress, neuroinflammatory cascades, and neurotransmitter deficits [11]. Traditional single-target synthetic drugs often fail to alter disease progression, driving intense research interest toward phenolic compounds [12]. These plant-derived metabolites offer distinct advantages due to their multi-target mechanisms of action, capable of simultaneously neutralizing reactive oxygen species (ROS) and regulating enzymatic pathways associated with cognitive decline. In regions of high botanical diversity like Bulgaria, where medicinal plants comprise approximately 20% of the native flora (~700 species) [13], utilizing this natural treasury remains a vital but underexplored strategy. While the widely distributed *P. officinalis* is established in Bulgarian folk medicine for its anti-inflammatory and expectorant properties [14], and stands out among native flora for its high phenolic content [15], numerous related taxa remain completely uninvestigated. This leaves a critical knowledge gap regarding the chemical and pharmacological evolutionary variations within regional *Pulmonaria* species.

To address this fundamental gap, this study conducts the first systematic investigation of *Pulmonaria rubra* Schott, an underexplored species with distinctive regional significance. Additionally, the in vitro antioxidant and neuroprotective potential of a MeOH extract from the aerial parts of the species was investigated, providing a foundational framework for its potential development as a natural therapeutic agent.

## 2. Results and Discussion

### 2.1. UHPLC-HRMS Identification of Secondary Metabolites

**Figure 1:**
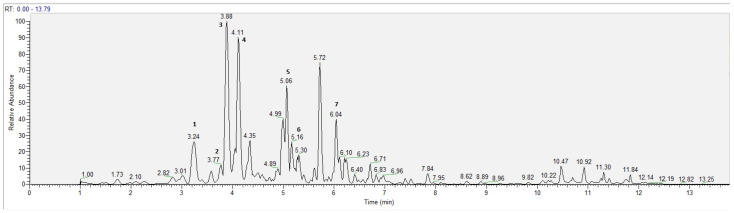
TIC chromatogram in the negative mode of fraction PR MCI 20.

**Figure 2:**
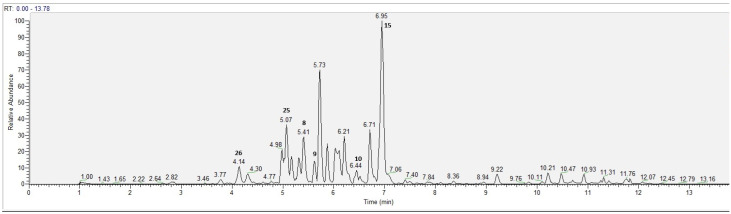
TIC chromatogram in the negative mode of fraction PR MCI 30.

**Figure 3:**
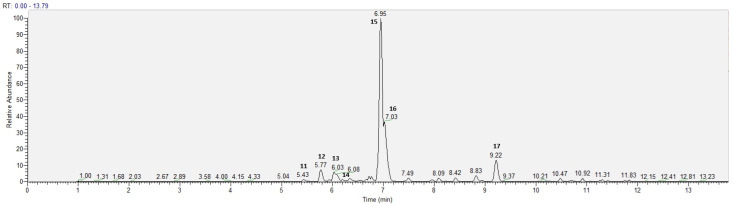
TIC chromatogram in the negative mode of fraction PR MCI 40.

**Figure 4:**
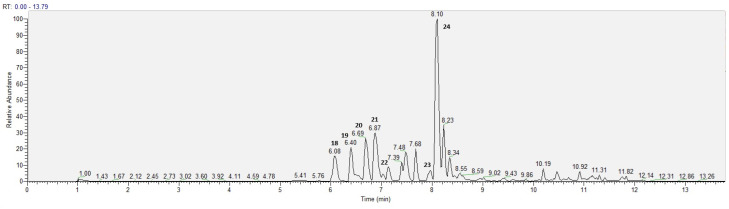
TIC chromatogram in the negative mode of fraction PR MCI 50.

The LC-MS/MS analysis of the MCI fractions from the defatted extract, using reference substances and database-assisted searches of the MS and MS/MS fragmentation patterns, led to the identification of 26 secondary metabolites (Table 1), primarily hydroxycinnamic acid derivatives and flavonoid glycosides. The method allowed for the determination of molecular formulae with mass errors generally below 3.0 ppm from the theoretical ones. Total ion current (TIC) chromatograms of the corresponding MCI gel fractions are presented in Figure 1, Figure 2, Figure 3 and Figure 4. Among the 26 identified metabolites, the hydroxycinnamic acid derivatives constituted the main chemical class, comprising 14 compounds (**1**–**10**, **15**–**17**, **23**–**24**), followed by 10 flavonoid glycosides (**11**–**14**, **18**–**22**, **25**) and two additional glycosides of phenolic and terpenoid origin (**2** and **26**). The majority of compounds were detected as deprotonated molecules [M-H]^−^ in negative ESI mode, while three compounds (**2**, **9**, **26**) were identified as formate adducts [M+HCOO]^−^. The observed mass errors across all identified metabolites ranged from 0.2 ppm (**21**, apigenin-hexoside) to 3.2 ppm (**13** and **23**), all well within the accepted ± 5 ppm threshold for high-resolution mass spectrometry, confirming the reliability of the molecular formula assignments. The distribution of identification confidence levels reflects the structural complexity of the metabolite pool: four compounds (**1**, **12**, **15**, **25**) were confirmed at Level 1 using authenticated reference standards, while the majority were assigned at Level 2a based on library-matched MS/MS spectra, with several flavonoid glycosides assigned at Level 2b due to the inherent inability of mass spectrometry to distinguish between hexose isomers.

#### 2.1.1. Hydroxycinnamic Acid Derivates and Caffeoyl Oligomers

The largest group of identified metabolites consists of hydroxycinnamic acid derivatives, primarily esters and oligomers of caffeic acid. In total, 14 compounds belonging to this class were identified, spanning simple caffeic acid esters (**3** and **4**), chlorogenic acid derivatives (**1**), complex caffeoyl oligomers (**5**–**10**), salvianolic acids (**16** and **23**), rosmarinic acid and its derivatives (**15** and **17**), and a lignan (compound **24**). Among these, caffeic acid esterification at different hydroxyl positions of small organic acids, threonic acid (**3**, *m*/*z* 297.0613) and glyceric acid (**4**, *m*/*z* 267.0508), were proved. The higher-order oligomers, including yunnaneic acids D (**6**, *m*/*z* 539.1198), E (**5**, *m*/*z* 571.1093), and F (**9**, *m*/*z* 597.1252), as well as methyl yunnaneate E (**7**, *m*/*z* 585.1254) and dihydrorabdosiin (**10**, *m*/*z* 719.1626), represent progressively larger polyphenolic assemblies built on rosmarinic acid and danshensu scaffolds. Lithospermic acid A (**8**, *m*/*z* 537.1038) and salvianolic acid A (**16**, *m*/*z* 493.1137) were characterized by the loss of danshensu residue, and, unlike the previous group of compounds, displayed a caffeic acid residue cleavage.

Compound **1** ([M-H]^−^ at *m*/*z* 353.0878, Figure S5) gave fragment ions at *m*/*z* 191.0554 [quinic acid–H]^−^ and *m*/*z* 179.0340 [caffeic acid–H]^−^ (Figure S6). Based on the higher relative abundance of the former ion [17], the compound was identified as neochlorogenic acid (5-*O*-caffeoylquinic acid). This phenolic acid has been recently reported to ameliorate Alzheimer’s disease-like pathology by scavenging oxidative stress and restoring blood–brain barrier function in zebrafish [18]. Compounds **3** and **4** were both identified as caffeic acid derivatives after database comparison and in accordance with the literature [6]. 2-*O*-(E)-caffeoyl-threonic acid identification was based on the parental ion [M-H]^−^ at *m*/*z* 297.0613 (Figure S9), together with the significant product ions at *m*/*z* 179.0342 [caffeic acid–H]^−^ and *m*/*z* 135.0286 (Figure S10), corresponding to [threonic acid–H]^−^. The MS/MS of compound **4** (*t*_R_ 4.11 min) revealed a fragment ion at *m*/*z* 105.0178 (Figure S12), which corresponded to [glyceric acid–H]^−^, allowing the compound to be identified as 2-*O*-(E)-caffeoyl-glyceric acid. Both acids have been previously identified in the aerial parts of *P. officinalis* [6].

The samples also contained complex caffeic acid oligomers, including Yunnaneic acid E (**5**), D (**6**), and F (**9**), as well as Methyl yunnaneate E (**7**). Yunnaneic acid E (*m*/*z* 571.1093, [M-H]^−^, Figure S13) was characterized by the diagnostic fragment at *m*/*z* 197.0455, representing the danshensu (3,4-dihydroxyphenyllactic acid) moiety, alongside a lithospermic acid B-type residue at *m*/*z* 285.0772 (Figure S14). In contrast, yunnaneic acid D (*m*/*z* 539.1195, [M-H]^−^, Figure S15) lacked the danshensu unit, instead yielding an intensive fragment with *m*/*z* 359.0777 (Figure S16), corresponding to a rosmarinic acid-like dimer residue, following the neutral loss of a caffeoyl unit. Yunnaneic acid F (**9**) was identified by its higher molecular weight precursor at *m*/*z* 597.1252 ([M-H]^−^, Figure S21), which, like compound **5**, produced an intensive danshensu fragment at *m*/*z* 197.0449 (Figure S22). All three compounds were first isolated from *Salvia yunnanensis* [19,20]. Similarly, Lithospermic acid A (**8**) (*m*/*z* 537.1038, [M-H]^−^, Figure S19) was identified by the loss of a danshensu unit to yield the fragment at *m*/*z* 295.0614, along with the diagnostic vinylcatechol ion at *m*/*z* 135.0438 (Figure S20).

Rosmarinic acid (**15**) (*m*/*z* 359.0771, [M-H]^−^, Figure S33) exhibited a characteristic fragmentation pattern with a base peak at *m*/*z* 161.0234 [caffeoyl–H_2_O]^−^ and an intensive fragment ion at *m*/*z* 197.0448 (Figure S34), representing the 3,4-dihydroxyphenyllactic acid moiety. Its derivative, Methyl rosmarinate (**17**) (*m*/*z* 373.0928, [M-H]^−^, Figure S37), was identified by the predominant ions at *m*/*z* 179.0341 [caffeic acid–H]^−^ and *m*/*z* 135.0439 (Figure S38). Salvianolic acid A (**16**) (*m*/*z* 493.1137, [M-H]^−^, Figure S35) was characterized by a highly stable fragment at *m*/*z* 295.0615 and a base peak at *m*/*z* 109.0281 (Figure S36), consistent with the loss of two caffeic acid residues. Salvianolic acids are known for their cardioprotective activities [21]. Among the larger polyphenolic structures, Dihydrorabdosiin (**10**) (*m*/*z* 719.1626, [M-H]^−^, Figure S23) showed a base peak at *m*/*z* 161.0234 and a dimer ion at *m*/*z* 359.0778 (Figure S24), indicating a caffeic acid tetramer. The compound has been identified in the roots of *Symphytum officinale*, which is most widely used in medicine plant from the Boraginaceae family [22,23]. Finally, the lignan Globoidnan A (**24**) (*m*/*z* 491.0981, [M-H]^−^, Figure S51) was identified by diagnostic ions at *m*/*z* 311.0564 and *m*/*z* 267.0663 (Figure S52), the latter representing a characteristic decarboxylation event within the lignan skeleton [22]. The compound has been firstly isolated from buds of *Eucalyptus globoidea* [24] and has been shown to exert in vitro antiproliferative, antioxidant and HIV-integrase inhibitory activity [24,25].

#### 2.1.2. Flavonoid Glycosides

Overall, 10 flavonoid glycosides were identified in *P. rubra* (compounds **11**–**14**, **18**–**22**, **25**), representing glycosylated derivatives of five distinct aglycones: quercetin, kaempferol, luteolin, apigenin, and hesperetin.

All quercetin glycosides were unified by the base peak at *m*/*z* 300.0278, corresponding to the [quercetin−H]^−^ aglycone radical, with additional characteristic A-ring and C-ring fragment ions at *m*/*z* 271.0250 and *m*/*z* 151.0026 arising from retro-Diels–Alder (RDA) fragmentation (Figures S28, S30 and S32), respectively, which are consistent with the established fragmentation patterns for flavonol-3-*O*-glycosides [26]. Rutin (**12**, *m*/*z* 609.1461) was confirmed by the loss of the rutinose moiety (glucosyl-rhamnosyl, 326 Da), whereas quercetin-hexoside (**13**, *m*/*z* 463.0882) showed only a hexose loss (162 Da). In addition, quercetin-malonylhexoside (**14**, *m*/*z* 549.0889) displayed not only hexose unit loss (162 Da) but a fragment corresponding to a malonyl moiety (86 Da). This structure was confirmed by the successive neutral losses of a CO_2_ (44 Da) and a loss of a ketene (CH_2_CO, 42 Da). Additionally, the rare quercetin triglycoside alcesefoliside (**25**, *m*/*z* 755.2050) was also identified. After the cleavage of the sugar chain (2,6-di-O-rhamnosyl-galactosyl, 455 Da), the quercetin aglycone was confirmed based on its characteristic fragment ions mentioned above for the aglycone (Figure S54) [27] in the MS/MS spectrum, and comparing to a reference substance.

Kaempferol glycosides (**19**, **20**, **22**) were analogously characterized by the [kaempferol−H]^−^ ion-radical at *m*/*z* 285.0405. Its flavonol structure was further confirmed by the characteristic A–C-ring fragment at *m*/*z* 151 (as a result of RDA fragmentation). Kaempferol-hexoside (**20**, *m*/*z* 447.0932) lost a hexose (162 Da, Figure S42), whereas kaempferol-methylpentosyl-hexoside (**19**, *m*/*z* 593.1512) showed a loss of a methylpentosyl-hexoside (326 Da) (Figure S44). The kaempferol-malonylhexoside (**22**, *m*/*z* 533.0940, Figure S48) was identified by the prominent loss of a hexose and a malonyl radical (as reported above for the quercetin derivative).

Hesperetin-methylpentosyl-hexoside (**11**) cleft the sugar unit (326 Da) and produced an aglycone fragment at *m*/*z* 300.0280 but without the characteristic for a flavonol fragment ion at *m*/*z* 255.0299 (Figure S25), confirming its structure. A luteolin-hexoside (**18**, *m*/*z* 447.0933) was also identified. The aglycone was at *m*/*z* 285.0406, following the cleavage of the hexose (162 Da). Luteolin was confirmed from a characteristic fragment ion at *m*/*z* 133.0282 [28] (Figure S40) in the MS/MS spectrum. In addition, the presence of apigenin-hexoside (**21**) was established as well, with a notable hexose moiety loss (162 Da). The aglycone (*m*/*z* 285.0377) is distinguished by an -OH (16 Da) from its counterpart luteolin. The MS/MS spectrum showed a specific fragment ion at *m*/*z* 117.0333.

Additionally, a putative myricetin-trihexoside (Figure 4, *t*_R_ 7.68 min) was detected (*m*/*z* 849.3614, [M+HCOO]^−^, Figure S57) based on sequential losses of three hexose units (3 × 162 Da) converging on an aglycone at *m*/*z* 317.1969 (myricetin, Figure S58), though formal assignment was not made.

The identified flavonoid pattern closely mirrors that reported for the related *P. officinalis* [2], providing phylogenetic coherence with the *Pulmonaria* genus.

#### 2.1.3. Phenolic and Terpenoid Glycosides

Two structurally distinct glycosides completed the metabolite profile of *P. rubra*: a phenolic glycoside of benzyl alcohol (**2**) and a C13-norisoprenoid glycoside, roseoside (**26**). Benzyl alcohol-pentosyl-hexoside (**2**) was detected as a formate adduct (*m*/*z* 447.1506) (Figure S7) and exhibited a characteristic sequential deglycosylation pattern in MS/MS: loss of the formate group (46 Da) to yield *m*/*z* 401.1457 (the deprotonated glycoside), followed by losses of the pentose (132 Da) and hexose (162 Da) moieties, with a diagnostic aglycone-hexoside fragment at *m*/*z* 269.1033 (Figure S8) [29]. Additionally, the C_13_-norisoprenoid roseoside (**26**) was also observed as a formate adduct at *m*/*z* 431.1920 (Figure S55). The MS/MS spectrum revealed a deprotonated molecular ion at *m*/*z* 385.1869. Subsequent MS/MS fragmentation provided a definitive structural fingerprint, characterized by the diagnostic neutral loss of a hexose unit (162 Da) to produce the deprotonated aglycone (vomifoliol) at *m*/*z* 223.1336. Further cross-ring cleavage of this core eliminated a side-chain fragment (70 Da), resulting in the highly stabilized cyclohexenone ring fragment at *m*/*z* 153.0909 (Figure S56) [30]. The compound has been first isolated from the aerial parts of *Catharantus roseus* (formerly *Vinca rosea*, Apocynaceae) [31]. So far, its presence was established in several species from different *Boraginaceae* genera [32,33,34] but this is the first report on the presence of this C_13_-norisoprenoid glycoside in a representative of the *Pulmonaria* genus. It has been previously shown to exhibit promising in vitro antitumour activity in a peroxynitrile-induced carcinogenesis [35], as well as antiviral effects against Hepatitis C virus [30]. Both compounds were present in minor quantities relative to the dominant hydroxycinnamic acid fraction, as reflected by their lower ion intensities in the TIC chromatograms (Figure 3 and Figure 4).

#### 2.1.4. Identification Confidence

Based on the Schymanski scale [16], several metabolites were identified as Level 1 (confirmed structure) and the majority of the compounds were identified at Level 2a (MS library match), as their accurate mass, MS/MS fragmentation patterns and relative ion abundance matched diagnostic data from the literature. Specifically, four compounds were assigned Level 1 (confirmed structure): neochlorogenic acid (**1**), rutin (**12**), rosmarinic acid (**15**), and alcesefoliside (**25**), each verified against co-injected certified reference standards. The majority of compounds (**3**–**10**, **16**–**17**, **23**–**24**, **26**) were assigned at Level 2a (putative identification) based on accurate mass measurements, library-matched MS/MS fragmentation patterns in mzCloud (https://www.mzcloud.org) and MassBank (https://massbank.eu), and concordance with the published literature. The remaining eight compounds (**2**, **11**, **13**, **14**, **18**–**22**) were assigned at Level 2b (tentative candidate), as their aglycone fragmentation was fully consistent with the proposed structures, but the identity of the attached sugar moiety could not be unambiguously confirmed by mass spectrometry alone due to the isobaric nature of hexose isomers (e.g., glucose vs. galactose, both 162 Da). The overall mass accuracy achieved across all 26 metabolites was excellent, with errors ranging from 0.2 to 3.2 ppm, all within the ± 5 ppm acceptance criterion commonly applied in HRMS-based metabolite identification [36]. These results collectively demonstrate the suitability of the applied UHPLC-HRMS workflow in negative ESI mode for the comprehensive profiling of polyphenolic and glycosidic secondary metabolites in *P. rubra*.

### 2.2. UHPLC-UV Identification of Phenolic Compounds

Using both *t*_R_ and UV maxima when identifying compounds with a reference substance, HPLC-UV is considered very precise. Often, glycosides of the same aglycone, substituted by the same sugar at different positions, have enough difference in the *t*_R_ to distinguish isomers [37]. Based on the information obtained from the LC-MS study, flavonoids and phenolic acids were selected to identify secondary metabolites in *P. rubra* by means of HPLC-UV.

Seven compounds were identified in the samples (Figures S1–S4) using reference substances, listed in Table 2. From the group of flavonoids, flavones and flavonols represent the most abundant subgroups of these metabolites and are very commonly distributed in overground parts of higher plants [38]. Among the compounds identified, apigenin-7-*O*-glucuronide and alcesefoliside were reported for the first time in genus *Pulmonaria*. Apigenin-7-*O*-glucuronide is believed to be an oxidative product of the corresponding hexoside, in this case the glucoside (also confirmed by reference substance) [39]. Alcesefoliside, a relatively rare secondary metabolite, has been previously shown to exert an in vivo neuroprotective effect against CCl_4_-induced brain toxicity in rats [40].

### 2.3. Quantitation of Rosmarinic Acid

**Figure 5:**
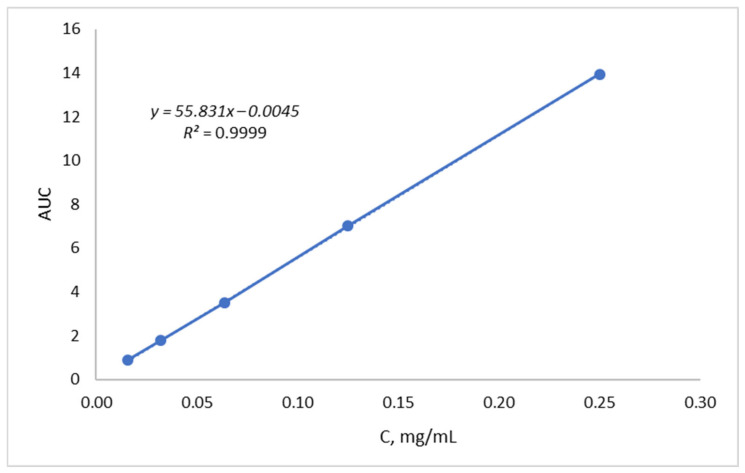
Calibration curve of RA.

From the UHPLC-HRMS, the main secondary metabolite identified in *P. rubra* was rosmarinic acid (RA), so an UHPLC-UV method for its determination was deployed. The calibration curve (Figure 5) of RA (see Section 3) gave a regression equation of *y* = 55.831*x* − 0.0045. The correlation coefficient (R^2^) of 0.9999 showed that the linearity of the method was excellent.

Since the method was optimized, and not newly developed, only partial validation was performed, concerning the linearity, limit of detection, and limit of quantitation [41].

**Figure 6:**
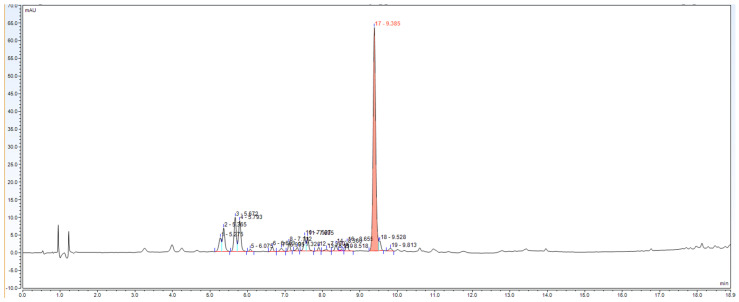
UHPLC-UV chromatogram of PRE with the peak corresponding to RA.

The method was linear from 0.016 to 0.250 mg/mL. RA retained a retention time under the specified chromatographic conditions (See Section 3) *t*_R_ = 9.362 ± 0.012 min. No other peaks were observed in the elution range of RA. After injection of the extract solution, a coincidence of *t*_R_ was established, which was 9.369 ± 0.018 min (average from three injections) (Figure 6).

The UV spectra of the compound in the sample coincided with those of the standard (329; 199). When a blank sample was injected, no peaks were observed in the elution range of the compound. From the regression equation, and the standard deviation (SD) of the lowest concentration’s response, the limit of detection (LOD) and the limit of quantitation (LOQ) were calculated using the equations LOD = 3.3 σ/S and LOQ = 10 σ/S, where σ is the SD and S is the slope of the calibration curve. The LOD was 0.0003 mg/mL and the LOQ was 0.0008 mg/mL. According to the ICH guideline, these are acceptable [42]. Moreover, the low value of the former gives the method’s excellent sensitivity. Since this concentration has no practical use for the studied samples, no experimental verification of the numerical value was performed. The calibration range does not extend down to the LOQ. Recovery was studied on model solutions of RA in the middle of the linear range (0.07 mg/mL). It was found that the mean recovery was 99.98% and the RSD was 1%.

Taking the sensitivity and the recovery of the quantitative method, it could be concluded that it was very useful [43]. In addition, the sample was introduced without any additional purification, resulting in short analysis time.

It was found that the quantity of RA in PRE was 4.35% (*w*/*w*), which is comparable to that in many species from the *Lamiaceae* family [44].

### 2.4. Pharmacological Investigation

The in vitro methods for determination of pharmacological activity play a key role in preliminary investigations of possible effects. Cellular and subcellular in vitro systems are of practical importance, since their structures represent the biochemical processes occurring in the cell [45]. Enzyme inhibition, as well as activation, are another key pharmacological mechanisms studied, often performed on recombinant enzymes [46]. Rat brain synaptosomes, mitochondria, and microsomes were used to investigate the PRE’s potential antioxidant and neuroprotective properties in vitro.

**Figure 7:**
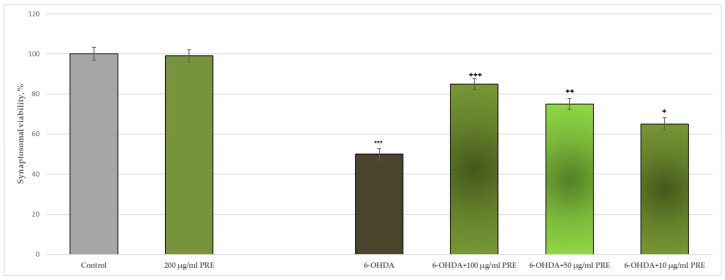
Effects of PRE on synaptosomal viability when administered alone and in an oxidative stress setting caused by 6-OHDA. *** *p* < 0.001 vs. untreated synaptosomes (control); ^+^ *p* < 0.05; ^++^ *p* < 0.01; ^+++^ *p* < 0.001 vs. 6-OHDA.

**Figure 8:**
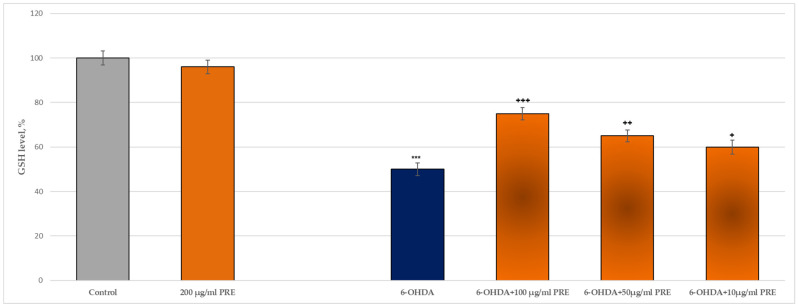
Effects of PRE in isolated synaptosomes, administered alone and in a model of 6-OHDA-induced oxidative stress on GSH level. *** *p* < 0.001 vs. untreated synaptosomes (control); ^+^ *p* < 0.05; ^++^ *p* < 0.01; ^+++^ *p* < 0.001 vs. 6-OHDA.

Administered alone, the PRE (200 μg/mL) had no pro-oxidative effect and did not influence the viability of the synaptosomes or their GSH levels (Figure 7 and Figure 8). 6-Hydroxydopamine (6-OHDA, 150 μM), as the toxic agent in this model, demonstrated statistically significant neurotoxic effects. In comparison to the untreated synaptosomes (control), it decreased GSH levels and synaptosomal viability by 50% (Figure 7 and Figure 8).

PRE treatment (10, 50, and 100 μg/mL) resulted in strong, concentration-dependent neuroprotective and antioxidant effects in the setting of 6-OHDA-induced oxidative stress, protecting synaptosomal viability and GSH levels at all tested concentrations. PRE showed the strongest effects on this model at the highest tested concentration (100 µg/mL) (Figure 7 and Figure 8).

Rat brain synaptosomes are an established and widely used subcellular model for investigating oxidative stress and neuroprotection in vitro, as they preserve the biochemical and bioenergetic properties of the presynaptic terminal [45]. In the present study, 6-OHDA was used as the neurotoxic agent to induce oxidative stress in isolated synaptosomes. The toxicity of 6-OHDA in this model is primarily attributed to its autooxidation and enzymatic metabolism to *p*-quinone, generating reactive oxygen species (ROS) including hydrogen peroxide, superoxide, and hydroxyl radicals. These ROS cause extensive lipid peroxidation, protein oxidation, and DNA damage, ultimately leading to mitochondrial dysfunction and neuronal cell death [47]. Accordingly, the capacity of a compound to counteract 6-OHDA-induced GSH depletion and viability loss in synaptosomes is indicative of genuine neuroprotective and antioxidant potential. Comparable concentration-dependent responses have been reported for other phenolic compounds. Encapsulated quercetin exerted pronounced neuroprotection in 6-OHDA-treated synaptosomes by stimulating non-enzymatic antioxidant defences and elevating GSH content [48]. The present neuroprotective effects can be attributed to the phenolic constituents of the **PRE**, which are known to scavenge ROS generated during 6-OHDA metabolism and to maintain endogenous GSH levels. This is in accordance with previous studies on flavonoid-rich extracts, where antioxidant protection was closely linked to the preservation of GSH [11,49].

**Figure 9:**
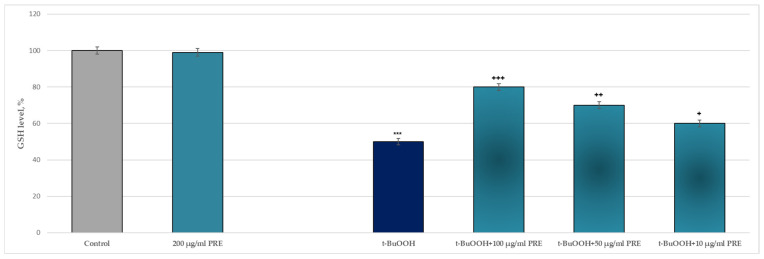
Effects of PRE on GSH level in brain mitochondria, administered alone and in a model of *t*-BuOOH-induced oxidative stress. *** *p* < 0.001 vs. control (untreated mitochondria); ^+^ *p* < 0.05; ^++^ *p* < 0.01; ^+++^ *p* < 0.001 vs. *t*-BuOOH.

**Figure 10:**
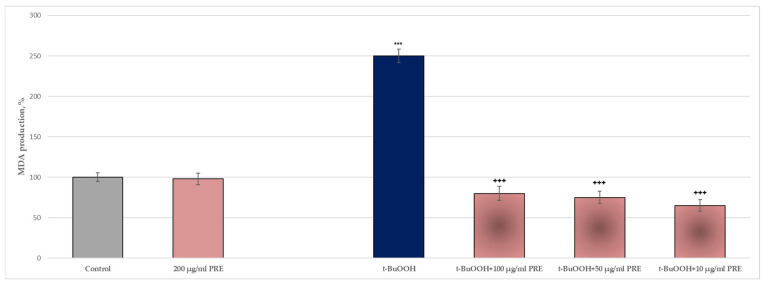
Effects of PRE administered alone and in a model of *t*-BuOOH-induced oxidative stress on MDA production in isolated brain mitochondria. *** *p* < 0.001 vs. control (untreated mitochondria); ^+++^ *p* < 0.001 vs. *t*-BuOOH.

When rat brain mitochondria were treated with PRE (200 μg/mL) alone, there was no statistically significant neurotoxic effect: neither the GSH level reduced, nor the MDA production increased. On the contrary, the toxic agent *tert*-butyl hydroperoxide (*t*-BuOOH, 75 μM), administered alone, decreased GSH levels by 50% and increased MDA generation by 150% in comparison to the untreated mitochondria (control), demonstrating a pro-oxidant and neurotoxic effect (Figure 9 and Figure 10).

In the *t*-BuOOH-induced oxidative stress setting, PRE demonstrated a strong, concentration-dependent neuroprotective and antioxidant effect on the isolated brain mitochondria (Figure 9 and Figure 10), resulting in a recovery of the GSH level and a reduction in MDA generation. Once more, the most effective concentration was the highest tested (100 µg/mL). The effects on MDA production were particularly noticeable, with an 80% decrease in its production.

Isolated rat brain mitochondria were used to assess the PRE’s protective capacity in a model of oxidative stress induced by *tert*-butyl hydroperoxide (*t*-BuOOH). *t*-BuOOH induces acute oxidative stress by forming free radical intermediates inside the cell via 2-electron oxidation and metal-ion-catalysed reactions, causing lipid peroxidation and depletion of reduced glutathione (GSH) [50]. Specifically, *t*-BuOOH is detoxified by GSH peroxidase, a reaction that consumes GSH and produces oxidised glutathione (GSSG). The resulting GSH depletion leads to loss of mitochondrial membrane integrity, impairment of respiratory chain function, and, ultimately, cell death [51]. The results from the current study corroborate previous ones [52], which demonstrated that intracerebroventricular injection of *t*-BuOOH produced a marked increase in cerebral GSSG levels (up to 90-fold) alongside a corresponding reduction in brain GSH—evidence of severe and rapid GSH oxidation. The subsequent increase in MDA observed in the present study is reflective of *t*-BuOOH-induced lipid peroxidation, consistent with the established mechanism of *t*-BuOOH toxicity [50]. These findings align with the neuroprotective profile reported on the same mitochondrial model [47]. The ability of the PRE to substantially reduce MDA—a secondary lipid peroxidation product and marker of oxidative damage—suggests that its phenolic constituents act as potent radical chain breakers, interrupting the peroxidation cascade at an early stage. Protection of the mitochondrial GSH pool is of particular significance, since mitochondria cannot efficiently export GSSG during oxidative stress, making them especially vulnerable to GSH depletion [51]. The antioxidant defence conferred by the PRE thus supports mitochondrial integrity and bioenergetics function under oxidative challenge.

**Figure 11:**
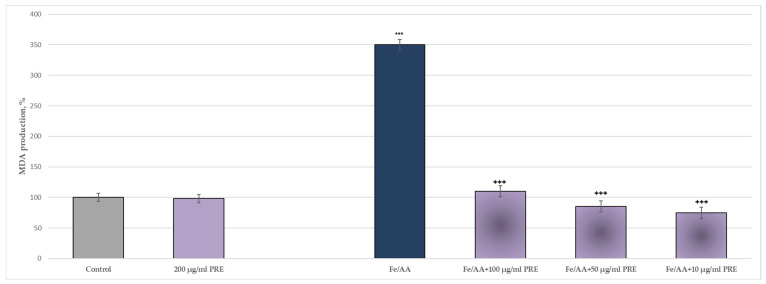
Effects of PRE administered alone and in a model of non-enzyme-induced lipid peroxidation on MDA production in isolated brain microsomes. *** *p* < 0.001 vs. control (untreated microsomes); ^+++^ *p* < 0.01 vs. Fe^2+^/AA.

On the isolated rat brain microsomes, in alone administration, PRE (200 μg/mL) had no statistically significant pro-oxidant effect (Figure 11). It did not influence the MDA production. When non-enzyme lipid peroxidation was induced with Fe^2+^/ascorbic acid (AA), it resulted in generating large amounts of MDA compared to the non-treated microsomes. When PRE was administered (10, 50, and 100 µg/mL), the MDA production percentage was much lower in the non-enzymatic lipid peroxidation conditions compared to the toxic agent (Fe^2+^/AA) (Figure 11).

Non-enzymatic lipid peroxidation was induced in isolated rat brain microsomes using a ferrous ion/ascorbic acid (Fe^2+^/AA) system. This model is well-established for assessing antioxidant activity in membrane-rich fractions: Fe^2+^ promotes the generation of superoxide anion radicals (O^2−•^) and hydroxyl radicals via the Fenton reaction, with ascorbic acid acting as a co-catalyst that continuously reduces Fe^3+^ back to Fe^2+^, thereby perpetuating the pro-oxidant cycle and leading to large amounts of MDA [12,53]. Accordingly, Fe^2+^/AA treatment of the microsomes in the present study generated a marked increase in MDA production relative to the untreated control. The protective mechanism of the PRE is consistent with the hydrogen-donating properties of phenolic compounds described in [12], which demonstrated that the inhibition of lipid peroxidation in Fe^2+^/AA-treated microsomes by phenolic compounds was primarily attributable to their radical-scavenging capacity rather than to iron chelation. This finding is yet confirmed by [54], which highlighted that phenolic compounds—including flavonoids—terminate lipid peroxidation chain reactions via electron or hydrogen atom donation, with their antioxidant potency strongly influenced by the hydroxylation pattern of the molecule. The diverse phenolic content of the PRE is therefore likely to account for its capacity to interrupt the free radical chain reaction initiated by Fe^2+^/AA in the microsomal membrane.

**Figure 12:**
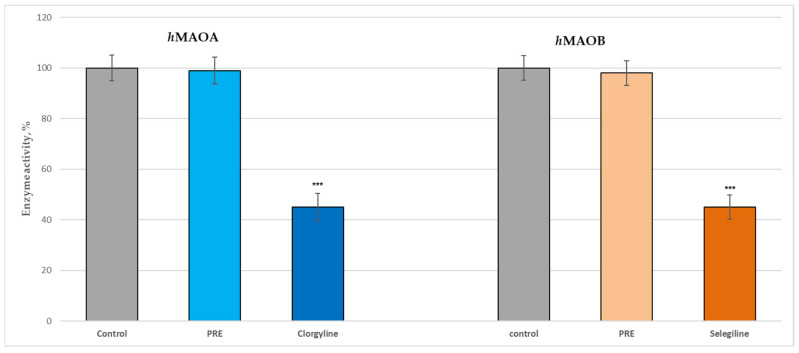
Effects of PRE on the *h*MAO-A and *h*MAO-*B* enzyme activity. *** *p* < 0.001 vs. control (untreated *h*MAO-A/B).

The PRE showed no impairment of the activity of the human recombinant MAO-A and MAO-B enzymes (*h*MAO-A/B). The traditional MAO-A and MAO-B inhibitors, chlorgyline and selegiline, reduced the enzyme activity by 45% when compared to the control (untreated enzyme) (Figure 12).

Monoamine oxidases A and B (MAO-A and MAO-B) are flavin adenine dinucleotide (FAD)-dependent enzymes bound to the outer mitochondrial membrane that catalyse the oxidative deamination of monoamine neurotransmitters, including dopamine, serotonin, noradrenaline, and adrenaline. Their inhibition is a validated pharmacological strategy for the management of Parkinson’s disease and depression, etc. [55]. However, MAO inhibition is not universally desirable in plant extracts intended for therapeutic development, as it may give rise to adverse drug interactions—particularly the tyramine-related hypertensive effect associated with irreversible MAO-A inhibitors [56]. This absence of MAO inhibitory activity indicates that the neuroprotective effects of the PRE are not mediated through monoamine oxidase inhibition but rather are attributable to its antioxidant and free radical-scavenging properties. This profile is of pharmacological interest. Not all phenol-rich plant extracts exhibit MAO inhibitory activity; several natural product extracts have been evaluated and found to lack significant MAO-A/B inhibitory effects, distinguishing their mechanisms from classical MAOI drugs [55]. By contrast, plant extracts demonstrating potent MAO inhibition—such as those from *Psoralea corylifolia* and *Ferula assafoetida* [56]—present a selective inhibitory profile. The absence of such activity in the PRE is therefore a favourable characteristic from a safety point, suggesting a lower possibility for tyramine-mediated hypertensive reactions compared to classical MAOIs. Furthermore, this result differentiates the PRE’s neuroprotective mechanism from those of flavonoid-rich extracts that are known inhibitors of human recombinant MAO, such as quercetin and myricetin (potent MAO-A inhibitors) or genistein (a selective MAO-B inhibitor) [57].

**Figure 13:**
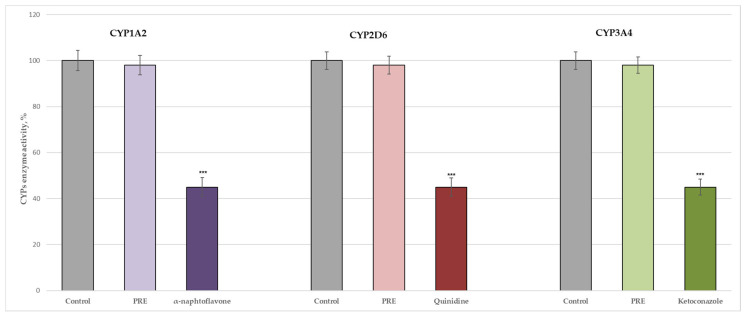
Effects of PRE on the enzyme activity of CYP1A2, CYP2D6 and CYP3A4. *** *p* < 0.001 vs. control.

PRE at a dose of 1 μg/mL did not show a statistically significant inhibitory effect on the activity of the enzymes CYP1A2, CYP2D6, and CYP3A4 as compared to the control (untreated isoforms (Figure 13)). The lack of interference with the CYP-mediated metabolic pathways suggests that the PRE has a favourable safety profile with a low potential for clinical drug–drug interactions [58]. These results support its potential for further development.

The cytochrome P450 (CYP) enzyme system is the primary mediator of oxidative drug metabolism in the liver. CYP1A2, CYP2D6, and CYP3A4 are among the most clinically relevant isoforms: together, they are responsible for the CYP-mediated metabolism of most clinically used drugs [59]. Inhibition of these enzymes by simultaneously administered substances—including herbal preparations—can lead to pharmacokinetic herb–drug interactions, resulting in elevated plasma drug concentrations, toxicity, or treatment failure [60]. Therefore, the assessment of the potential of a plant extract to inhibit CYP isoforms is a critical component of its preclinical safety evaluation. The results indicate that the PRE, at the tested concentration, is unlikely to interfere with the CYP-mediated metabolic pathways responsible for the biotransformation of a wide range of co-administered drugs, conferring a favourable clinical safety profile with low potential for drug–drug interactions. This finding contrasts with the profile of several plant extracts that are established CYP inhibitors. The extract of *Hypericum perforatum* is a well-documented inducer of CYP3A4, lowering the plasma concentrations of co-administered drugs. Similarly, grapefruit juice furanocoumarins potently inhibit CYP3A4 and other isoforms [61]. Various medicinal plant extracts—including those from *Hyptis verticillata*, *Spirostachys africana*, and *Moringa oleifera*—have been shown to produce moderate-to-potent inhibition of CYP1A2, CYP2D6, and/or CYP3A4 in in vitro settings [59,62]. The absence of significant CYP inhibitory activity by the PRE thus represents a meaningful advantage over extracts with known interaction potential, supporting its further development.

These results support the antioxidant and neuroprotective in vitro effects of PRE, which are attributed to its diverse phenolic content. Given that rosmarinic acid was identified as one of the primary constituents in the plant extract, it is highly probable that the observed neuroprotective activity is largely mediated by this compound. RA has been extensively documented to possess robust antioxidant, anti-inflammatory, and antiapoptotic properties [63]. It exerts these protective effects by directly scavenging ROS, alleviating mitochondrial dysfunction, and modulating key cell signaling pathways such as NF-κB and Nrf2 [64]. Corroborating these mechanisms, an in vivo study using a 6-OHDA-induced rat model demonstrated that intragastric administration of RA (20 mg/kg) exerts a neuroreparative function on the degenerating nigrostriatal dopaminergic system, specifically by decreasing nigral iron levels and regulating Bcl-2/Bax gene expression [65]. Consequently, the substantial presence of this compound likely underpins the extract’s ability to preserve neuronal viability and defend against neurotoxic damage.

## 3. Materials and Methods

### 3.1. General

Ultra-pure water was prepared by Millipore Milli-Q^®^ purification system (Merck, Darmstadt, Germany). UHPLC-gradient grade MeCN, MeOH and trifluoroacetic acid (TFA) were purchased from Merck (Darmstadt, Germany). TLC analysis was performed on Kieselgel F_254_ plates (Merck, Darmstadt, Germany), developed in a solvent system of EtOAc:EtCOMe:HCOOH:H_2_O (5:3:1:1) and visualized with Naturstoff reagent A (diphenylboric acid 2-aminoehyl ester solution in MeOH, 1%), then observed under UV light (366 nm).

### 3.2. Extraction and Fractionation

The overground parts of *P. rubra* were harvested on 15 April 2024 from Vitosha Mt., Bulgaria (42°36′52.5″ N 23°14′01.7″ E). One of the authors (I.S.) confirmed the identity of the species. A voucher specimen (SOM-179530) is kept at the Herbarium of the Institute of Biodiversity and Ecosystem Research, Bulgarian Academy of Sciences. After the air-dried plant material (24 g) was ground into a 3 mm powder, the lipophilic components were extracted using CH_2_Cl_2_ (6 × 250 mL) and sonication (37 kHz, 800 W, 30 min/each). After airing in the fume cupboard, the defatted plant material was extracted using sonication (37 kHz, 800 W, 30 min/each) with 80% MeOH (6 × 400 mL), then the methanol extracts were pooled, filtered, evaporated under vacuum and then lyophilised. A dry extract (5.8 g) was produced and named PRE. The extract was separated using medium-pressure column chromatography over MCI™ gel (Mitsubishi Chemical Corporation, Chiyoda, Japan), eluting with H_2_O:MeOH (0 → 100%). Eight main fractions were collected (PR MCI 0–100). TLC analysis with Naturstoff reagent A revealed that the fractions PR MCI 20, 30, 40 and 50 were rich in phenolic compounds.

### 3.3. UHPLC-HRESIMS Identification

A Q Exactive™ Plus Orbitrap^©^ mass spectrometer equipped with a hot electrospray ionisation (HESI) ion source (ThermoFisher Scientific, Bremen, Germany) was linked to a UHPLC system (Dionex UltiMate™ 3000 RSLC, ThermoFisher Scientific, Germering, Germany). The full-scan MS had a resolution of 70,000 (at 200 *m*/*z*), an AGC target of 3e^6^, a maximum IT of 100 ms, and a scan range of 250 to 1700 *m*/*z*. The ion source operated at −2.5 kV and 320 °C (capillary and probe), with an S-Lens RF level of 50.0, 38 arbitrary units (a.u.) of sheath gas and 12 a.u. of auxiliary gas (both N_2_), according to the program Tune^©^ (ThermoFisher Scientific, Bremen, Germany). A Kromasil^®^ C_18_ column (1.9 μm, 2.1 × 50 mm, Akzo Nobel, Bohus, Sweden) was used. It was maintained at 40 °C, and eluted (0.3 mL/min) with (A) 0.1% HCOOH in H_2_O and (B) 0.1% HCOOH in MeCN. The gradient program consisted of 0.5 min of 10% B, 7 min of increasing to 30% B, 1.5 min of 30% B (isocratic), 3.5 min of increasing to 95% B, 2 min of 95% B (isocratic), and 0.1 min back to 10% B. Each fraction was dissolved in MeOH (0.02 mg/mL), filtered through a 0.22 µm PVDF filter and injected to the UHPLC. The software Xcalibur^©^, version 4.2 (Thermo Scientific, Bremen, Germany) was used to collect raw data and to process the results. For the identification of secondary metabolites, the m/zCloud (https://www.mzcloud.org) and MassBank (https://massbank.eu) databases were used. The mass accuracy was set to 5 ppm. The observed accurate mass, MS/MS fragmentation patterns and relative ion abundance were used to compare with database information.

### 3.4. UHPLC-UV Identification and Assay

A second UHPLC system (Vanquish™, ThermoFisher Scientific, Germering, Germany) with a diode array detector was used. A Symmetry^®^ C_18_ column (3.5 μm, 4.6 × 75 mm, Waters, Drinagh, Ireland) was used, maintained at 35 °C, and eluted with H_2_O + 0.1% TFA (A) and MeCN (B) with a constant flowrate of 0.8 mL/min. The gradient program was initial 10% B, from 0 to 25 min—10%, to 95% B, with a 5-min re-equilibration before the next sample. The UV spectrum of each peak was recorded in the interval of 190–860 nm [44]. For identification purposes, each fraction was dissolved in MeOH (10 mg/mL), and filtered (PVDF, 0.22 µm), and 2 µL were injected three times to obtain mean *t*_R_. Reference substances, listed in Table 2 (purity > 99%, HPLC), purchased from Sigma-Aldrich (Tauchkirchen, Germany), were used for this identification. Compounds were identified by matching their retention times (*t*_R_) and UV–vis spectra [39] with those of the reference compounds (Table 2).

For the quantitative analysis, a stock solution of RA was prepared in methanol (1 mg/mL). Work solutions were made from it separately with concentrations as follows: 0.016, 0.032, 0.064, 0.125 and 0.250 mg/mL, in the same solvent, sealed in autosampler vials, and immediately used. Two μL of each solution were injected to obtain the chromatogram. From three replicates (*n* = 3), the mean retention time and the mean AUC were calculated for each concentration. PRE was dissolved in MeOH (5 mg/mL), and filtered through a PVDF filter (0.22 µm), and 2 µL were injected to determine the quantity of RA. All injections were in triplicate. For the quantification, the analytical wavelength was 330 nm. The software Chromeleon™ (v. 7.3.2, Thermo Fisher Scientific, Germering, Germany) was used to collect data, to construct the calibration curve, and to perform quantitation.

### 3.5. In Vitro Pharmacological Evaluation

For the isolation of the subcellular structures, frozen (−40 °C) rat brain was used. It was homogenized after defrosting using a Teflon pestle with the appropriate buffers, as described in the procedures below. Percoll reagent was utilized for the gradient centrifugation. Work solutions (16, 10, and 7.5%) were made from a stock solution of 90% Percoll and employed as explained below. Synaptosomes, microsomes, and mitochondria were immediately harvested and then treated for 1 h with varying doses of *Pulmonaria rubra* extract (PRE) solution in DMSO.

Using Percoll gradient, repeated subcellular fractionation was used to prepare the synaptosomes from rat brain homogenate. PRE alone, 150 μM 6-OHDA, or a combination of 6-OHDA and PRE, were used to incubate the synaptosomes for 1 h. An MTT-test was used to assess synaptosomal viability, and spectrophotometry was used to measure the amount of GSH in the synaptosomes [66].

By centrifuging the brain homogenate twice at 100,000× *g* for 1 h, microsomes were obtained. Twenty μM FeSO_4_ and 0.5 mM ascorbic acid (AA) were used to initiate Fe^2+^/AA-induced lipid peroxidation (LPO). PRE was added both by itself and in various concentrations under LPO conditions. The amount of MDA, a lipid peroxidation product, was measured [66].

The mitochondria were isolated through multiple rounds of differential fractionation. PRE was evaluated both on its own and under conditions of oxidative stress. Induction of oxidative stress in the mitochondria was achieved by incubating them with 75 μM *tert*-butyl hydroperoxide (*t*-BuOOH). Following the incubation, the total amount of malondialdehyde (MDA) produced in each sample was assessed, along with the measurement of glutathione (GSH) levels within the mitochondria [66].

Using the Amplex UltraRed-reagent fluorimetric method [46], the investigated extract was assessed for potential inhibitory activity on the human recombinant (*h*) MAO-A and MAO-B enzymes (Abcam, Cambridge, UK). The enzymes were treated with the PRE (1 µM in DMSO) for 1 h.

The activity of three isoforms of human recombinant cytochrome P450 (CYP1A2, CYP3A4, and CYP2D6) was assessed using a fluorimetric technique [67]. Inhibitor screening kits were purchased from Affigen (Vancouver, BC, Canada). The isoforms were treated with the PRE (1 µM in DMSO) for 2 h.

The statistical software “Medcalc” v. 18 (MedCalc Software Ltd., Ostend, Belgium) was used to analyze the data. Every experiment was conducted three times, and the results are shown as the mean of three (*n* = 3). The results were examined for statistical significance using a Mann–Whitney non-parametric test. The differences were deemed significant when *p* < 0.05, *p* < 0.01, or *p* < 0.001.

## 4. Conclusions

The presented study hereby provides the first comprehensive evaluation of the neuroprotective and antioxidant potential of Pulmonaria rubra Schott, establishing its biological efficacy alongside its traditionally significant relative, *P. officinalis*. The results demonstrate that the methanol extract (PRE) exerts significant concentration-dependent antioxidant effects in various in vitro rat brain models, effectively mitigating oxidative stress induced by 6-hydroxydopamine and *tert*-butyl hydroperoxide. Crucially, this robust neuroprotection occurs without any interference with CYP450 isoforms under the tested conditions, pointing to a highly favorable safety profile and low risk of plant–drug interactions. Phytochemical profiling via UHPLC-MS and UHPLC-UV of PRE revealed the presence of 26 secondary metabolites, notably hydroxycinnamic acids, caffeoyl oligomers, and flavonoids. A key finding of this research was the identification of apigenin-7-O-glucuronide, alcesefoliside and roseoside, reported here for the first time within the genus Pulmonaria, expanding the chemotaxonomic understanding of these plants. Furthermore, the high content of rosmarinic acid (4.35%) position *P. rubra* as an exceptionally potent, high-yield natural source of this therapeutic phenolic compound. These findings support the further in vivo evaluation of *P. rubra*, laying the groundwork for targeted neuroprotective drug development.

## Figures and Tables

**Table 1:** Compounds identified in *P. rubra* by UHPLC-HRMS, negative ESI.

No.	Compound Name	Theoretical Formula	Observed Ion Formula	Error (ppm)	Observed Ion, *m*/*z*	Ion Type	Major Fragments, *m*/*z* (% Intensity)	Level *
1	Neochlorogenic acid (5-*O*-(*E*)-caffeoylquinic acid)	C_16_H_18_O_9_	C_16_H_17_O_9_^−^	1.5	353.0878	[M-H]^−^	191.0554 (100), 179.0340 (2), 85.0280 (12)	1
2	Benzyl alcohol-pentosyl-hexoside	C_18_H_26_O_10_	C_19_H_27_O_12_^−^	2.5	447.1506	[M+HCOO]^−^	269.1033 (100), 401.1457 (45), 71.0123 (43)	2b
3	2-*O*-(*E*)-caffeoyl-threonic acid	C_13_H_14_O_8_	C_13_H_13_O_8_^−^	1	297.0613	[M-H]^−^	135.0286 (100), 179.0342 (5), 161.0235 (3)	2a
4	2-*O*-(*E*)-caffeoyl-glyceric acid	C_12_H_12_O_7_	C_12_H_11_O_7_^−^	1.5	267.0508	[M-H]^−^	105.0178 (100), 161.0233 (34)	2a
5	Yunnaneic acid E	C_27_H_24_O_14_	C_27_H_23_O_14_^−^	1	571.1093	[M-H]^−^	135.0439 (100), 285.0772 (43), 241.0868 (53)	2a
6	Yunnaneic acid D	C_27_H_24_O_12_	C_27_H_23_O_12_^−^	1.1	539.1198	[M-H]^−^	135.0439 (100), 161.0233 (76), 179.0341 (33), 359.0777 (13)	2a
7	Methyl yunnaneate E	C_28_H_26_O_14_	C_28_H_25_O_14_^−^	1.2	585.1254	[M-H]^−^	135.0439 (100), 311.0563 (31), 299.0940 (7)	2a
8	Lithospermic acid A	C_27_H_22_O_12_	C_27_H_21_O_12_^−^	0.9	537.1038	[M-H]^−^	135.0438 (100), 295.0614 (53), 185.0235 (8)	2a
9	Yunnaneic acid F	C_29_H_26_O_14_	C_29_H_25_O_14_^−^	1.3	597.1252	[M-H]^−^	135.0439 (100), 197.0449 (21), 179.0342 (15)	2a
10	Dihydrorabdosiin	C_36_H_32_O_16_	C_36_H_31_O_16_^−^	1.9	719.1626	[M-H]^−^	161.0234 (100), 359.0778 (31), 197.0448 (17)	2a
11	Hesperetin-methylpentosyl-hexoside	C_27_H_30_O_16_	C_27_H_29_O_16_^−^	1.8	609.1466	[M-H]^−^	300.0280 (100), 271.0252 (42)	2b
12	Rutin	C_27_H_30_O_16_	C_27_H_29_O_16_^−^	0.9	609.1461	[M-H]^−^	300.0278 (100), 271.0250 (55), 255.0299 (23), 151.0026 (14)	1
13	Quercetin-hexoside	C_21_H_20_O_12_	C_21_H_19_O_12_^−^	3.2	463.0882	[M-H]^−^	300.0279 (100), 271.0251 (50)	2b
14	Quercetin-malonylhexoside	C_24_H_22_O_15_	C_24_H_21_O_15_^−^	1.4	549.0889	[M-H]^−^	300.0278 (100), 271.0251 (41), 505.0994 (48)	2b
15	Rosmarinic acid	C_18_H_16_O_8_	C_18_H_15_O_8_^−^	1.4	359.0771	[M-H]^−^	161.0234 (100), 197.0448 (22), 179.0342 (13)	1
16	Salvianolic acid A	C_26_H_22_O_10_	C_26_H_21_O_10_^−^	0.6	493.1137	[M-H]^−^	109.0281 (100), 295.0615 (85), 185.0237 (60)	2a
17	Methyl rosmarinate	C_19_H_18_O_8_	C_19_H_17_O_8_^−^	1.3	373.0928	[M-H]^−^	135.0439 (100), 179.0341 (81)	2a
18	Luteolin-hexoside	C_21_H_20_O_11_	C_21_H_19_O_11_^−^	1.1	447.0933	[M-H]^−^	285.0406 (100), 151.0026 (5), 133.0281(5)	2b
19	Kaempferol-methylpentosyl-hexoside	C_27_H_30_O_15_	C_27_H_29_O_15_^−^	1	593.1512	[M-H]^−^	285.0405 (100), 255.0300 (42), 227.0347 (32)	2b
20	Kaempferol-hexoside	C_21_H_20_O_11_	C_21_H_19_O_11_^−^	1.6	447.0932	[M-H]^−^	285.0406 (100), 151.0026 (5)	2b
21	Apigenin-hexoside	C_21_H_20_O_10_	C_21_H_19_O_10_^−^	0.2	431.0981	[M-H]^−^	268.0377 (100), 151.0022 (3), 117.0333 (1), 107.0122 (3)	2b
22	Kaempferol-malonyl-hexoside	C_24_H_22_O_14_	C_24_H_21_O_14_^−^	1.7	533.0940	[M-H]^−^	285.0404 (100), 489.1041 (31), 255.0290 (49), 227.0346 (31)	2b
23	Methyl ester of Salvianolic acid H	C_28_H_24_O_12_	C_28_H_23_O_12_^−^	3.2	551.1204	[M-H]^−^	135.0438 (100), 161.0232 (98), 193.0498 (78), 359.0778 (14)	2a
24	Globoidnan A	C_26_H_20_O_10_	C_26_H_19_O_10_^−^	0.6	491.0981	[M-H]^−^	311.0564 (100), 267.0663 (29), 135.0439 (45)	2a
25	Alcesefoliside	C_33_H_40_O_20_	C_33_H_39_O_20_^−^	2.1	755.2050	[M-H]^−^	300.0277 (100), 271.0249 (58), 255.0298 (28), 151.0023 (6)	1
26	Roseoside	C_19_H_30_O_8_	C_20_H_31_O_10_^−^	2.3	431.1923	[M+HCOO]^−^	153.0909 (100), 385.1869 (80), 223.1336 (55)	2a

* According to Schymanski’s scale [16].

**Table 2:** Compounds identified in *P. rubra* using UHPLC-UV with reference substances.

Compound	*t*_R_ (min) ± SD	UV_max_, nm	Present in PRE
Spiraeoside	8.812 ± 0.103	366; 253	−
Luteolin	11.358 ± 0.154	348; 253	−
Isorhamnetin	13.743 ± 0.142	371; 254	−
Gossypin	9.355 ± 0.161	377; 258	−
Apigenin	13.398 ± 0.132	337; 267	−
Kaempferol	13.617 ± 0.112	366; 265	−
Chrysin	15.120 ± 0.122	313; 268	−
Alcesefoliside	13.445 ± 0.120	355; 255	+
Luteolin-7-*O*-glucuronide	7.795 ± 0.101	348; 254	−
Luteolin-3’-*O*-glucuronide	9.350 ± 0.151	340; 268	−
Orientin	6.53 ± 0.142	349; 256	−
Kaempferol-3-*O*-glucoside (Astragalin)	8.342 ± 0.139	347; 265	+
Robinin	6.292 ± 0.103	347; 265	−
Apigenin-7-*O*-glucuronide	9.037 ± 0.099	337; 266	+
Apigenin-7-*O*-glucoside	8.705 ± 0.124	337; 266	+
Vitexin	7.300 ± 0.113	337; 267	−
Quercetin-3-*O*-glucoside (Isoquercetin)	7.605 ± 0.118	354; 256	+
Quercitrin	8.510 ± 0.158	349; 256	−
Rutin	7.187 ± 0.130	354; 256	+
Rhamnetin	14.502 ± 0.120	371; 256	−
Myricitrin	7.433 ± 0.113	350; 260	−
Verbascoside	7.617 ± 0.145	330; 218	−
Eriodyctiol	11.415 ± 0.126	288; 199	−
Camelliaside A	6.262 ± 0.127	347; 265	−
Rosmarinic acid	9.362 ± 0.099	329; 199	+
Chlorogenic acid	4.215 ± 0.101	326; 195	−

## Data Availability

Data connected with this study are freely available from the corresponding author, upon reasonable written request. The data is not publicly available due to the large volume of the files.

## Supplementary Materials

The following supporting information can be downloaded at: https://www.mdpi.com/article/10.3390/ijms27146122/s1.

## Abbreviations

The following abbreviations are used in this manuscript:

PRE	*Pulmonaria rubra* extract
UHPLC	Ultra-high pressure liquid chromatography
UV	Ultraviolet
MS	Mass spectrometry
*h*MAO-A/B	Human recombinant monoaminoxidase type A/B
COX	Cyclooxygenase
MPLC	Medium-pressure liquid chromatography
TLC	Thin-layer chromatography
6-OHDA	6-hydroxydopamine
*t*-BuOOH	*tert*-butyl hydroxyperoxide
ISK	Inhibitor screening kits
MDA	Malondialdehyde
EDTA	Ethylenediaminetetraacetic acid
GSH	Reduced glutathione
MTT	3-(4,5-dimethylthiazol-2-yl)-2,5-diphenyltetrazolium
HEPES	4-(2-hydroxyethyl)-1-piperazineethanesulfonic acid
TFA	Trifluoroacetic acid
AGC	Automatic gain control
HESI	Heated electrospray ionisation
CRS	Chemical reference substance
RDA	Retro-Diels–Alder
HR-MS	High-resolution mass spectrometry
NMR	Nuclear magnetic resonance
RA	Rosmarinic acid
AA	Ascorbic acid
TIC	Total ion current
