# Supplementary material for: Unveiling Pulmonaria rubra Schott: Phytochemical Characterisation and Evaluation of Its Neuroprotective Potential
Source: Int J Mol Sci. 2026 Jul 8;27(14):6122. doi: 10.3390/ijms27146122 (PMC13409941; doi:10.3390/ijms27146122)
Supplement: Supplementary file 1 [file ijms-27-06122-s001.zip › ijms-4395560-supplementary.pdf]

International Journal of
Molecular Sciences

MDPI

Article

Unveiling *Pulmonaria rubra* Schott: Phytochemical Characterisation and Evaluation of Its Neuroprotective Potential

Ivan Stambolov[1], Aleksandar Shkondrov[1]*, Lyubomira Vusheva[1], Magdalena Kondeva-Burdina[2] and Ilina Krasteva[1]

[1] Department of Pharmacognosy, Faculty of Pharmacy, Medical University of Sofia, 2 Dunav st., 1000 Sofia, Bulgaria; istambolov@pharmfac.mu-sofia.bg (I.S.); shkondrov@pharmfac.mu-sofia.bg (A.S.); 105230@students.mu-sofia.bg (L.V.); ikrasteva@pharmfac.mu-sofia.bg (I.K.)
[2] Laboratory of Drug Metabolism and Drug Toxicity, Department of Pharmacology, Pharmacotherapy and Toxicology, Faculty of Pharmacy, Medical University of Sofia, 2 Dunav st., 1000 Sofia, Bulgaria; mkondeva@pharmfac.mu-sofia.bg (M.K.B.)

*Correspondence: shkondrov@pharmfac.mu-sofia.bg

Supplementary material

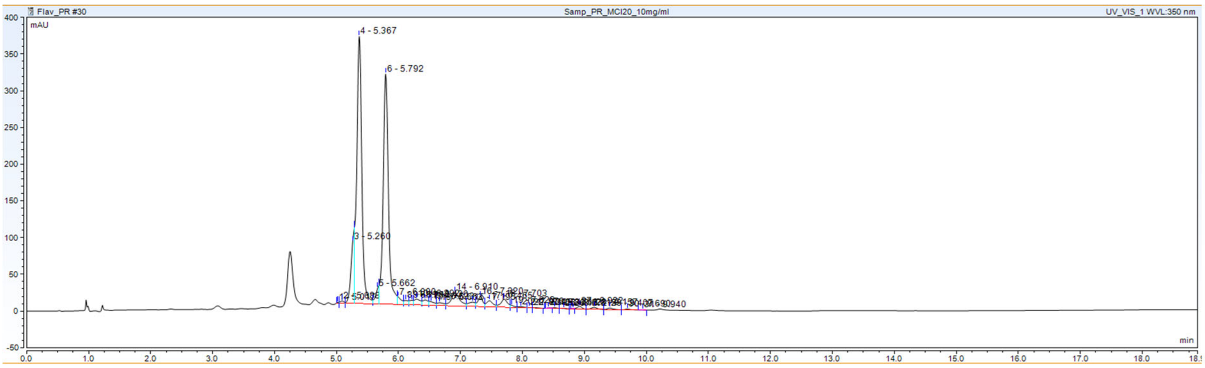

Figure S1. UHPLC-UV chromatogram of fraction PR MCI 20

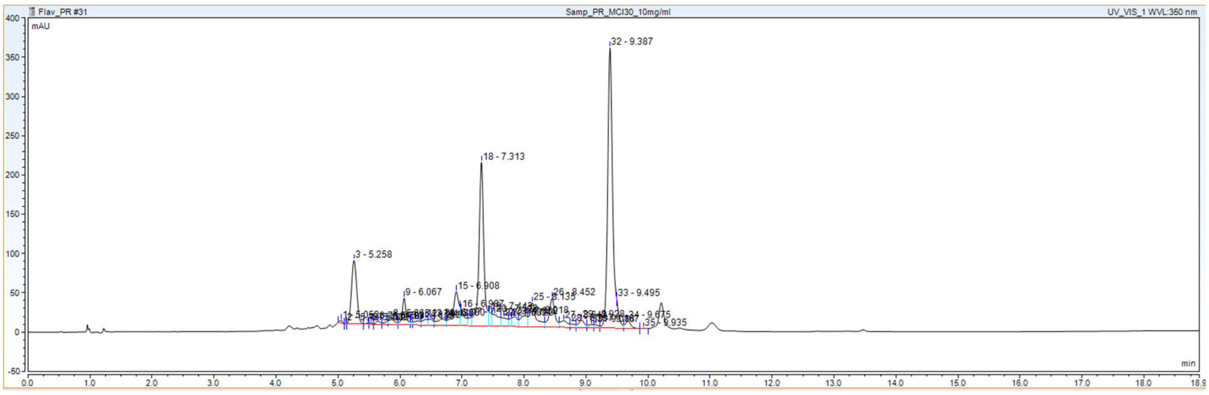

Figure S2. UHPLC-UV chromatogram of fraction PR MCI 30

 https://doi.org/10.3390/ijms27146122

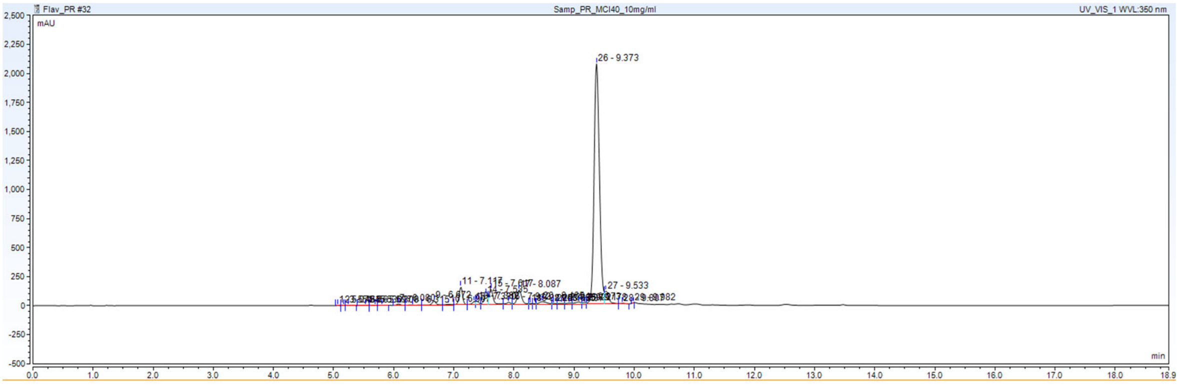

Figure S3. UHPLC-UV chromatogram of fraction PR MCI 40

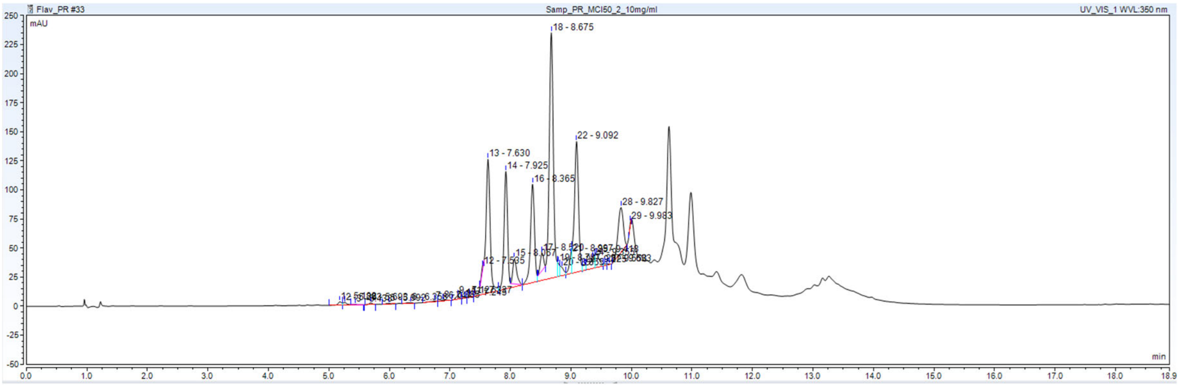

Figure S4. UHPLC-UV chromatogram of fraction PR MCI 50

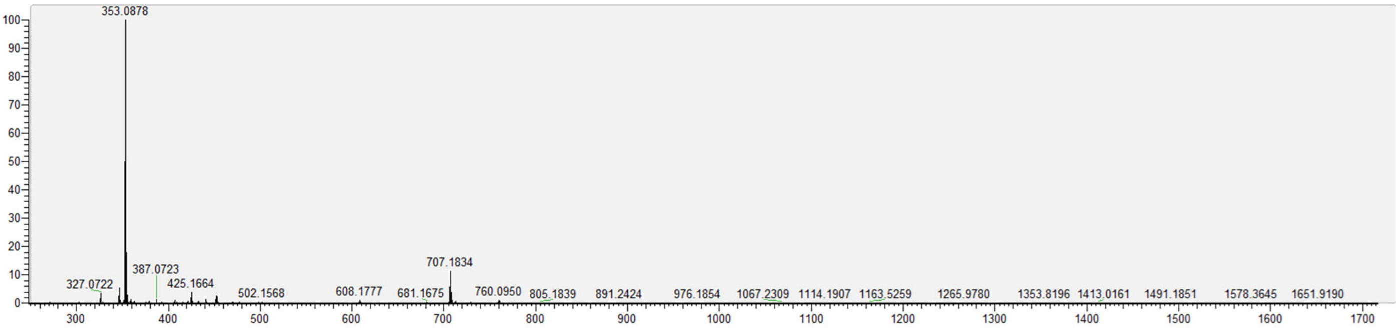

Figure S5. MS spectrum of Neochlorogenic acid

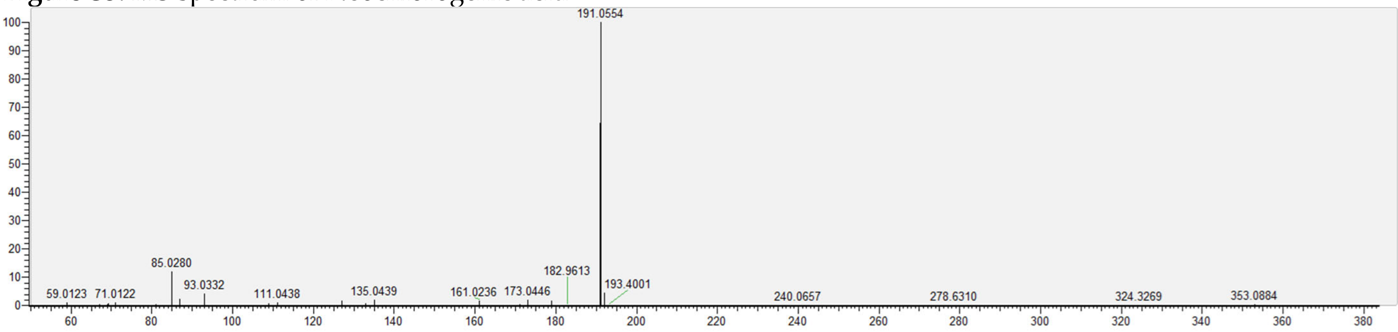

Figure S6. MS^2 spectrum of Neochlorogenic acid

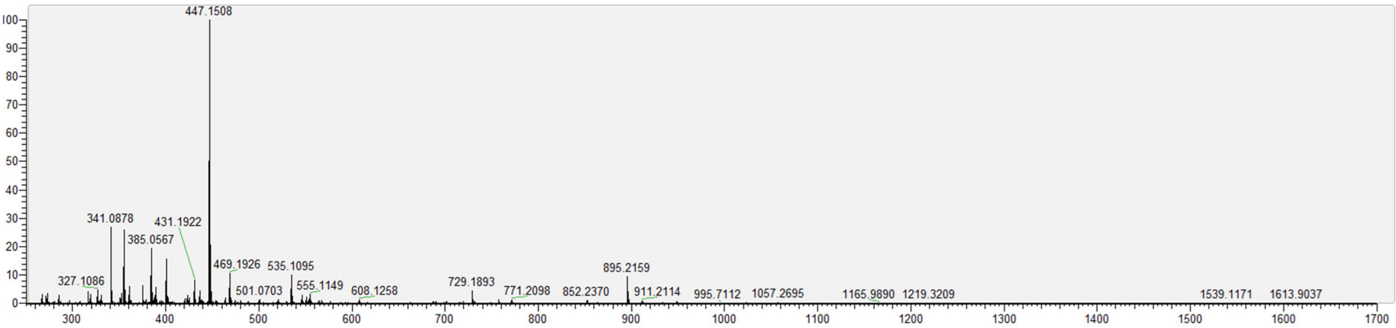

Figure S7. MS spectrum of Benzyl alcohol-pentosyl-hexoside

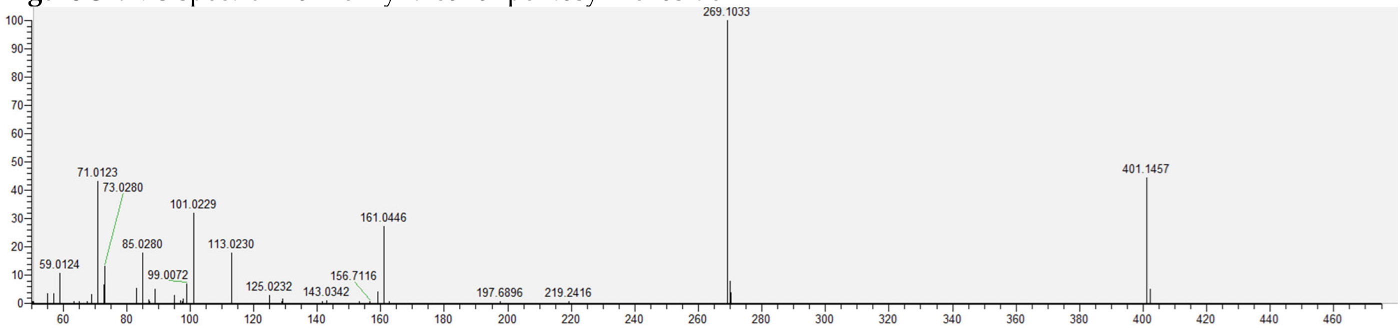

Figure S8. MS² spectrum of Benzyl alcohol-pentosyl-hexoside

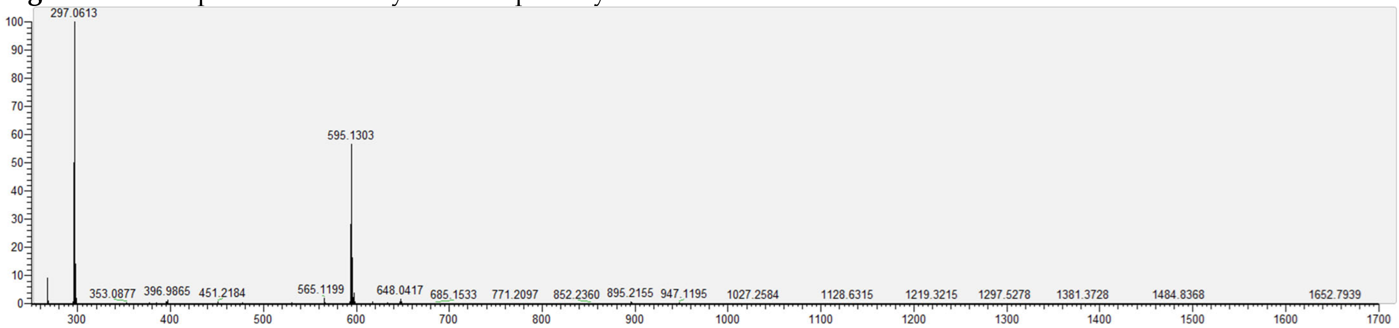

Figure S9. MS spectrum of 2-*O*-(*E*)-caffeoyl-threonic acid

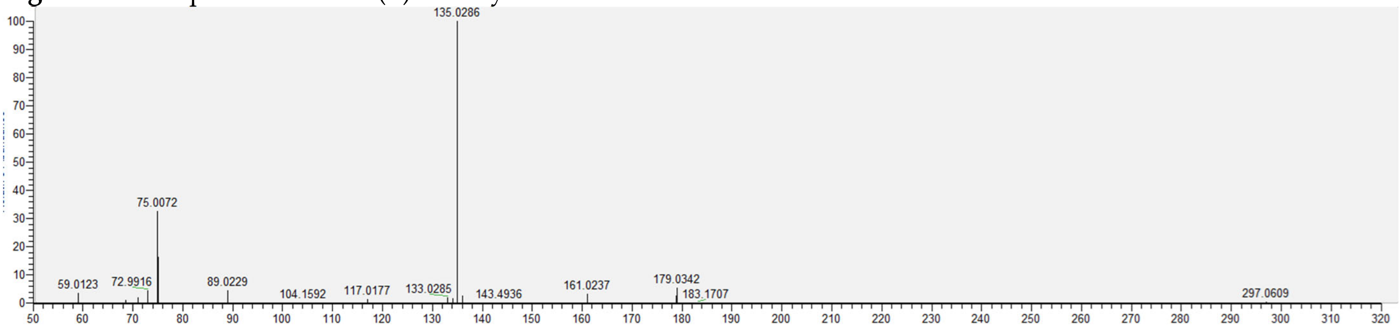

Figure S10. MS² spectrum of 2-*O*-(*E*)-caffeoyl-threonic acid

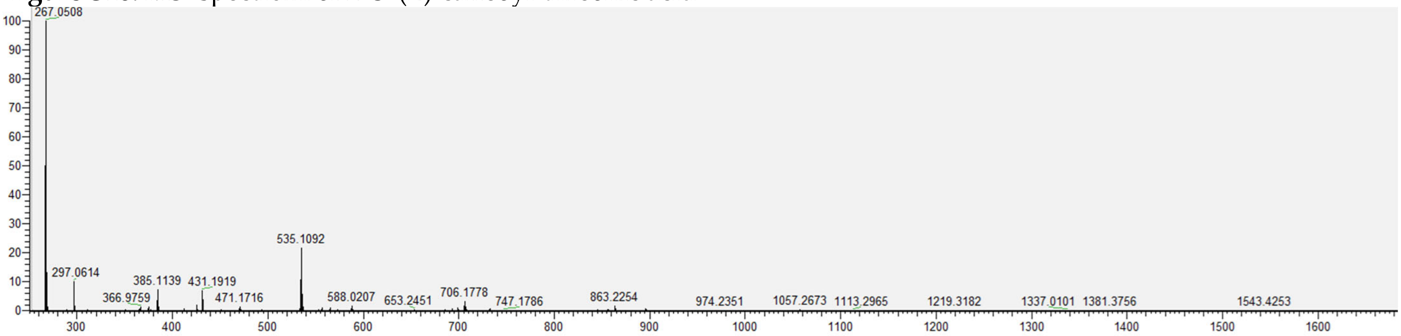

Figure S11. MS spectrum of 2-*O*-(*E*)-caffeoyl-glyceric acid

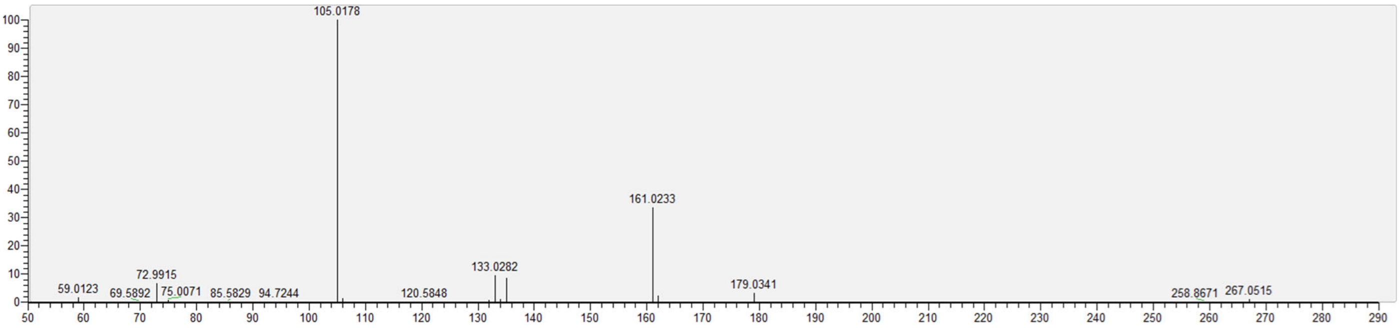

Figure S12. MS² spectrum of 2-*O*-(*E*)-caffeoyl-glyceric acid

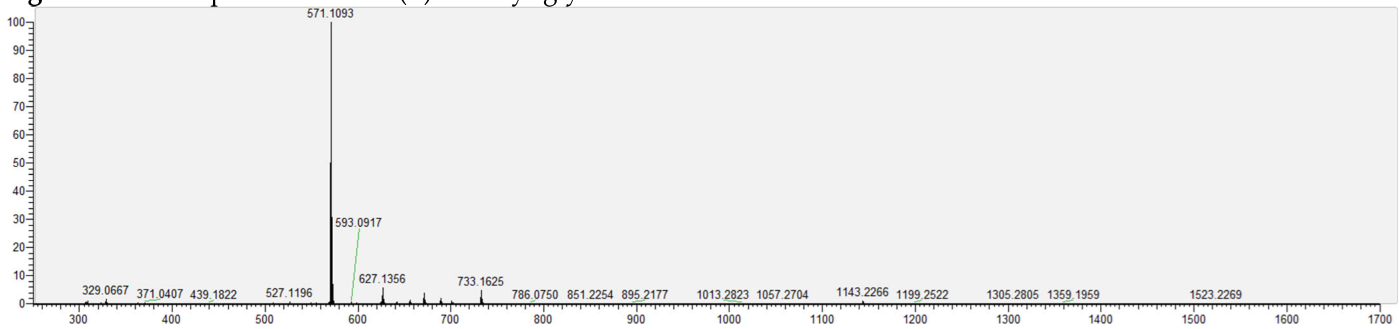

Figure S13. MS spectrum of Yunnaneic acid E

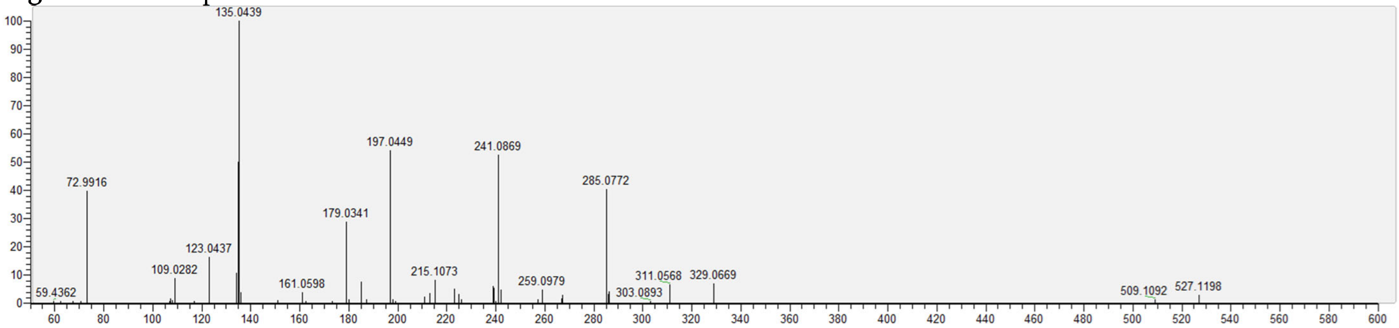

Figure S14. MS² spectrum of Yunnaneic acid E

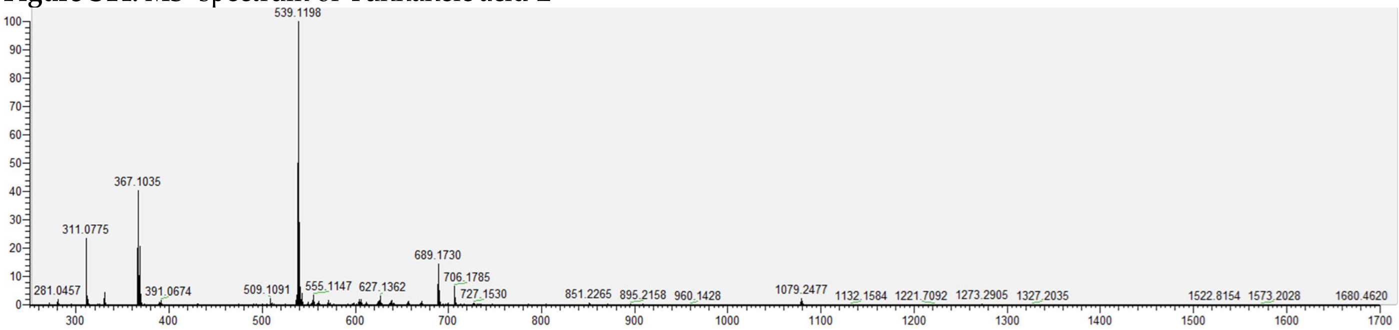

Figure S15. MS spectrum of Yunnaneic acid D

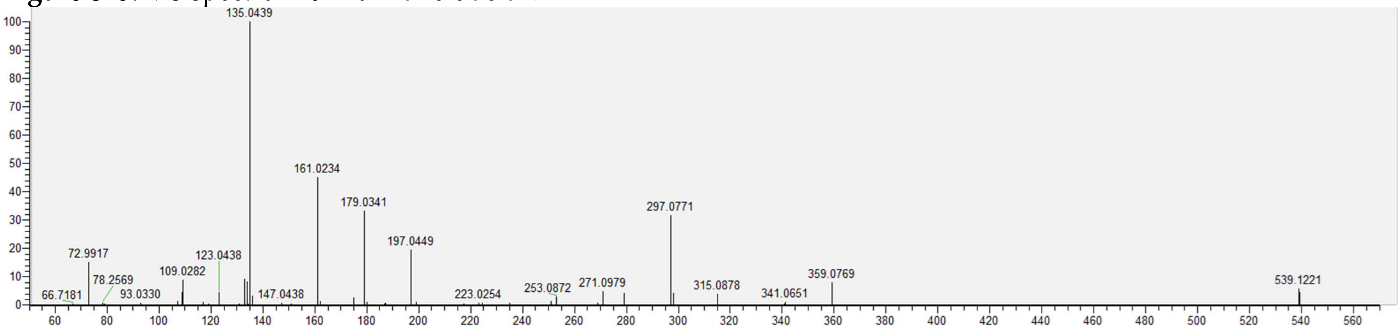

Figure S16. MS² spectrum of Yunnaneic acid D

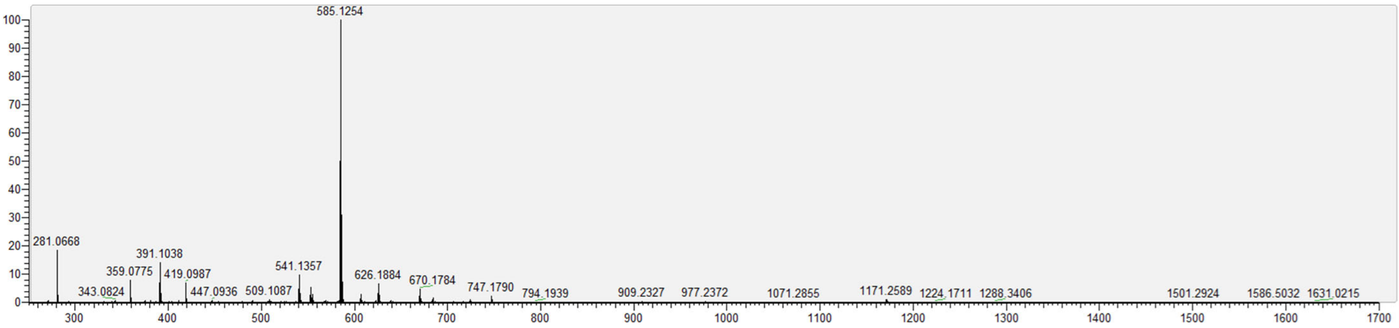

Figure S17. MS spectrum of Methyl yunnaneate E

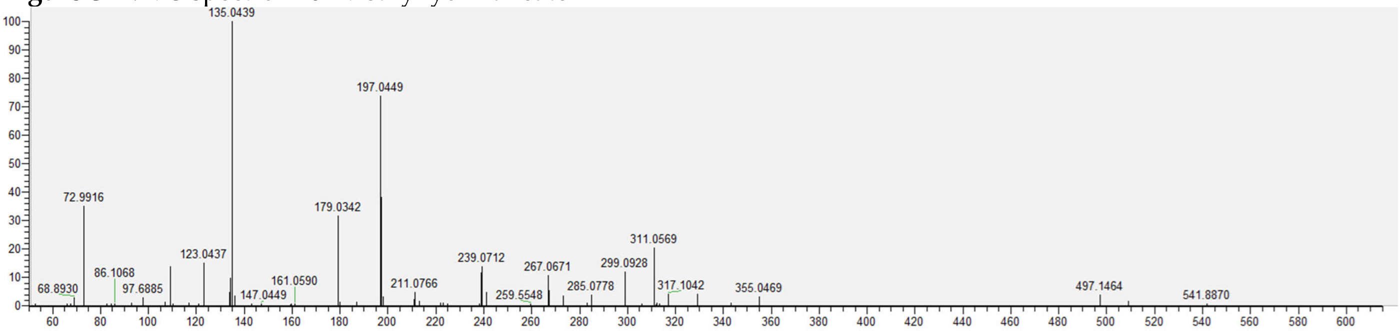

Figure S18. MS² spectrum of Methyl yunnaneate E

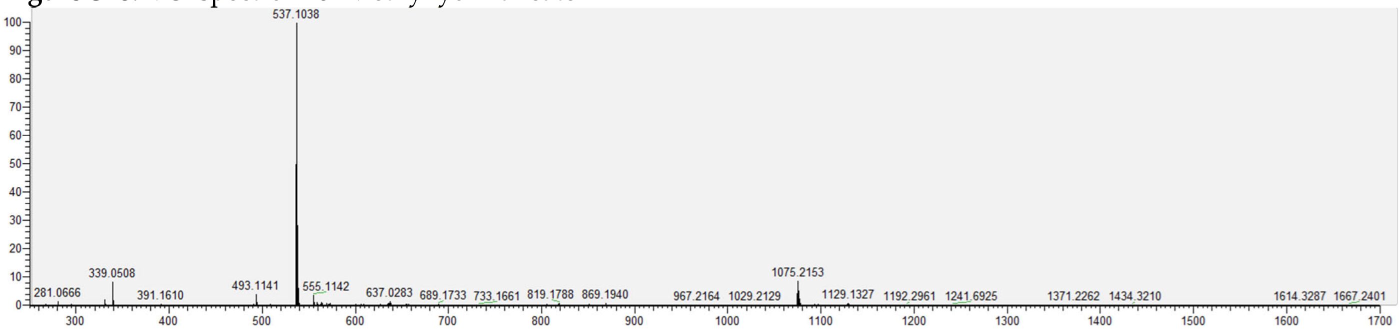

Figure S19. MS spectrum of Lithospermic acid A

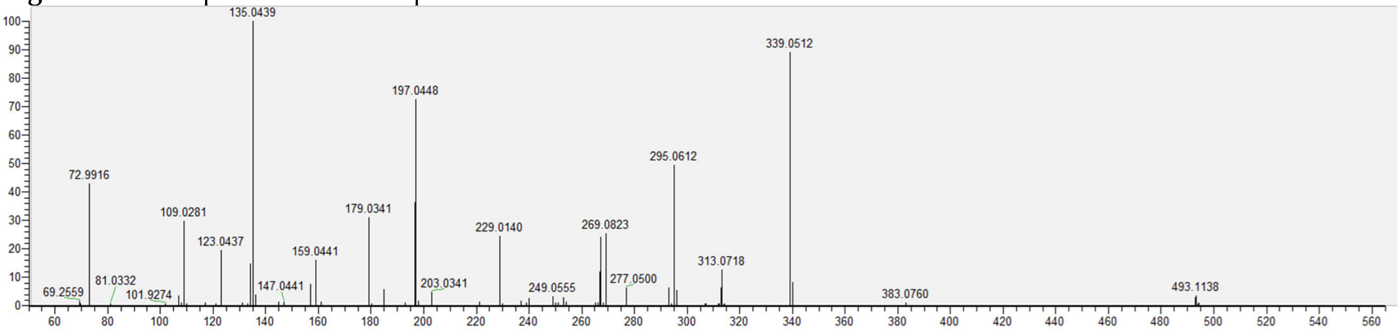

Figure S20. MS² spectrum of Lithospermic acid A

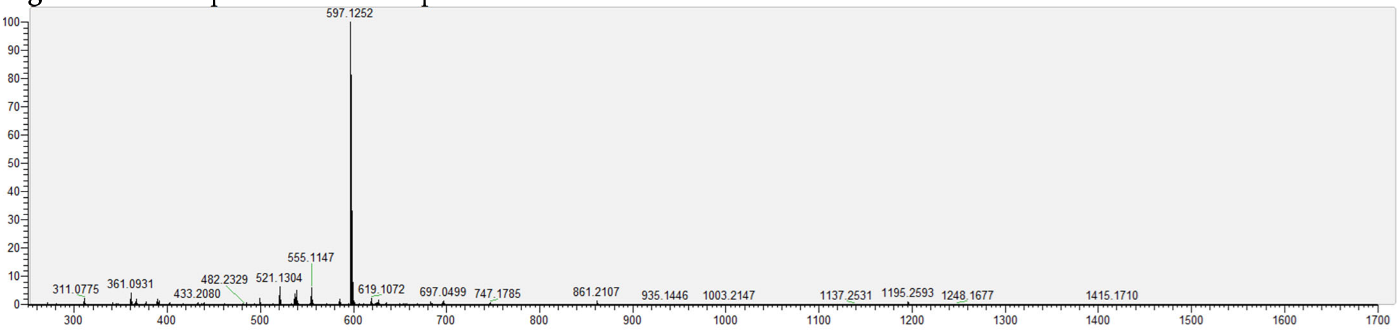

Figure S21. MS spectrum of Yunnaneic acid F

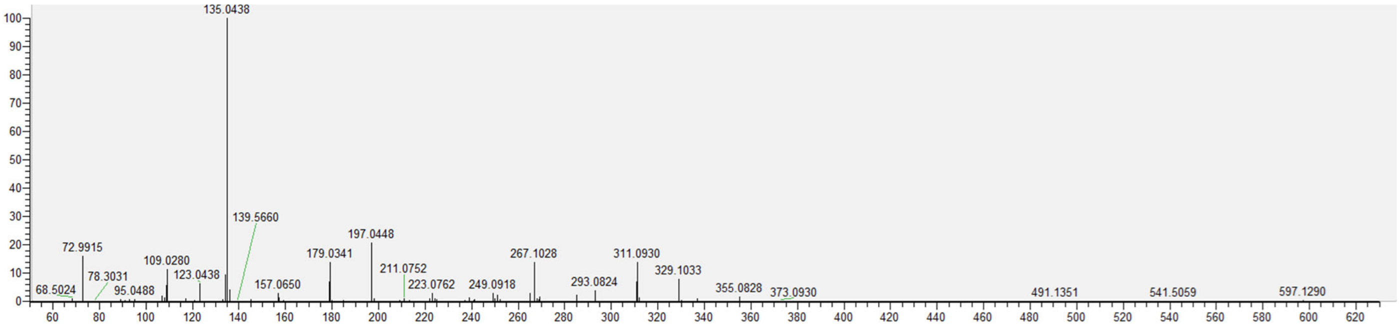

Figure S22. MS^2 spectrum of Yunnaneic acid F

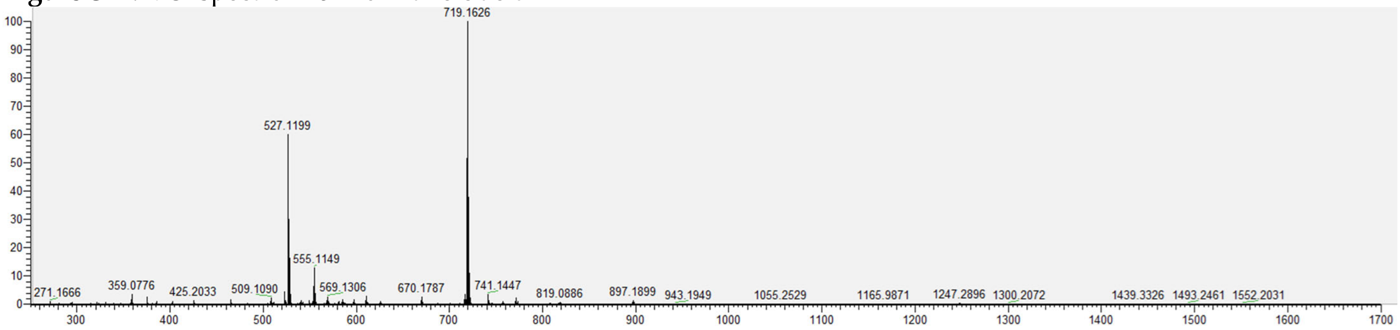

Figure S23. MS spectrum of Dihydrorabdosiin

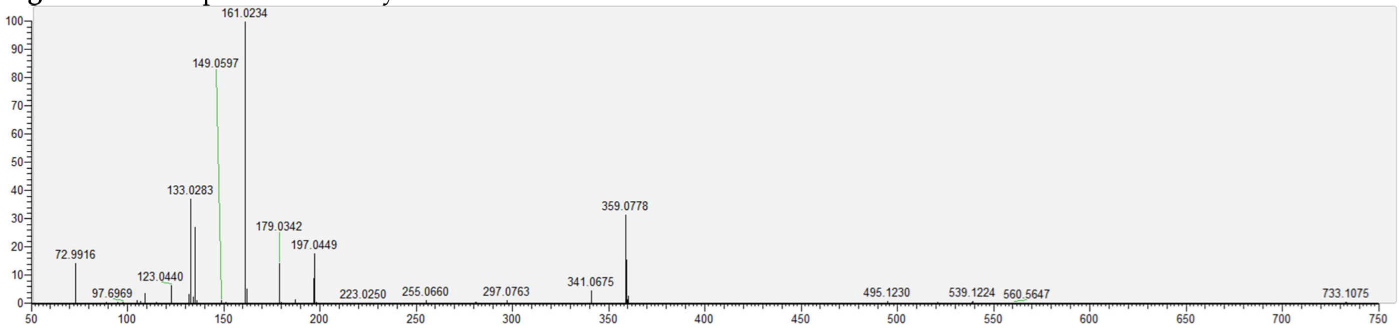

Figure S24. MS^2 spectrum of Dihydrorabdosiin

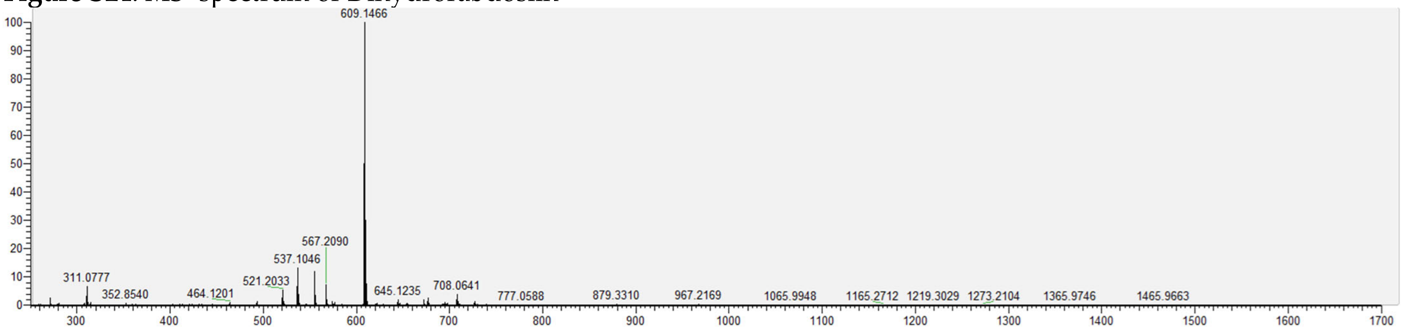

Figure S25. MS spectrum of Hesperetin-methylpentosyl-hexoside

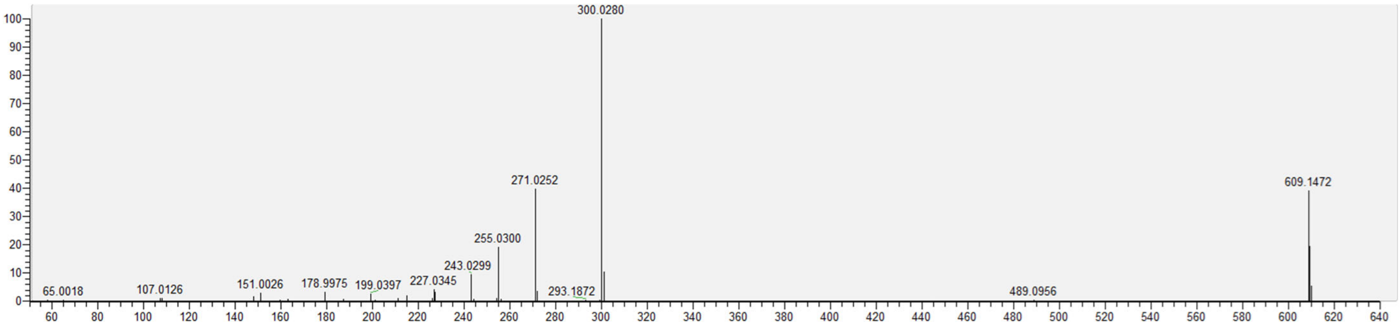

Figure S26. MS^2 spectrum of Hesperetin-methylpentosyl-hexoside

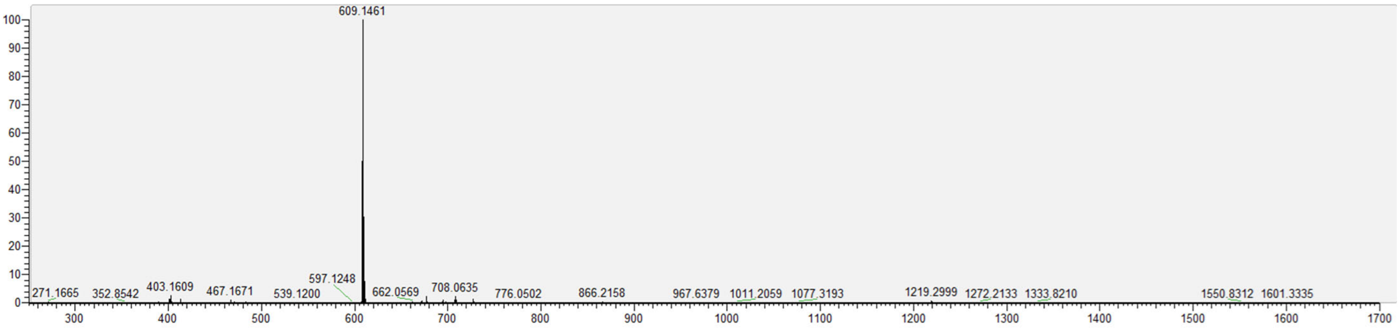

Figure S27. MS spectrum of Rutin

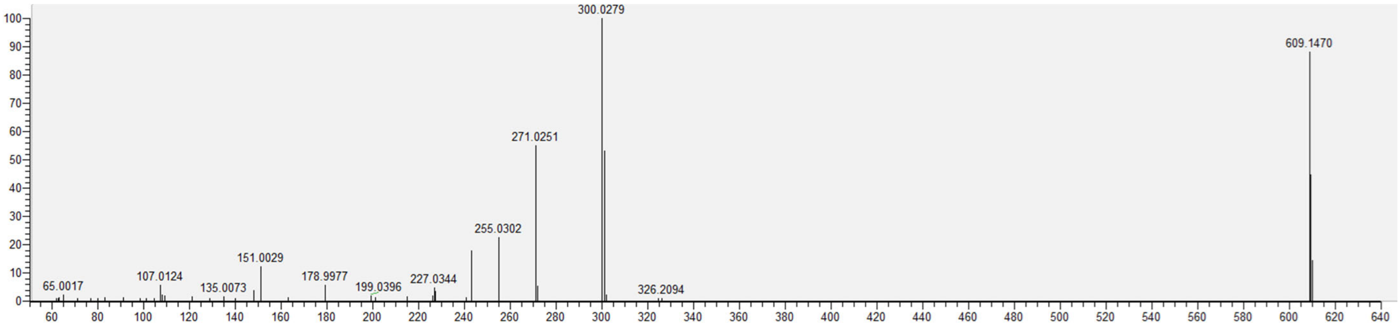

Figure S28. MS^2 spectrum of Rutin

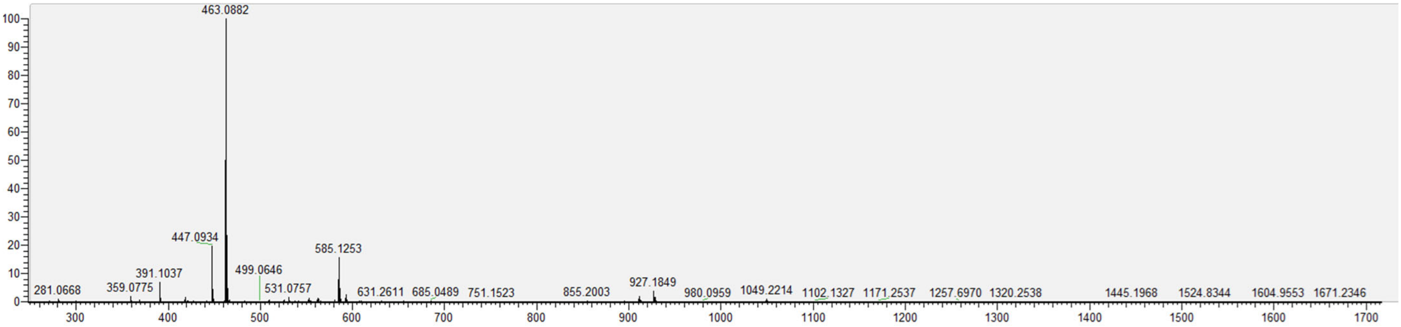

Figure S29. MS spectrum of Quercetin-hexoside

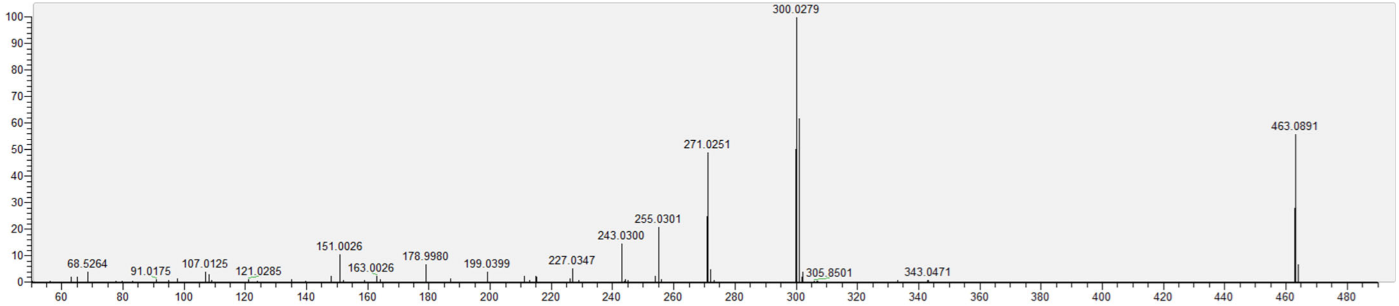

Figure S30. MS^2 spectrum of Quercetin-hexoside

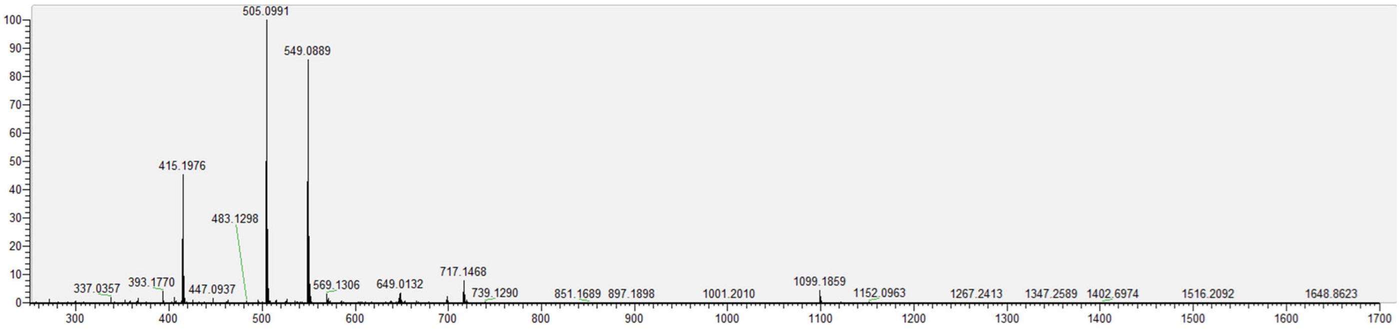

Figure S31. MS spectrum of Quercetin-malonylhexoside

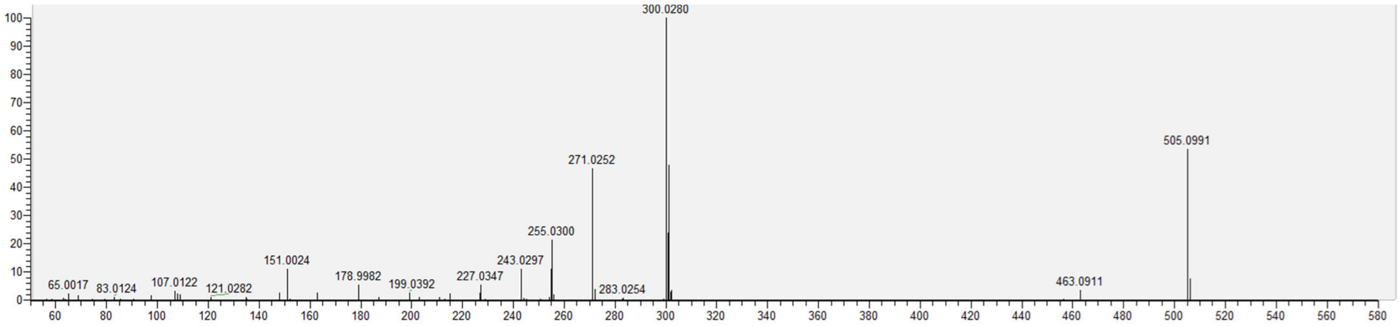

Figure S32. MS² spectrum of Quercetin-malonylhexoside

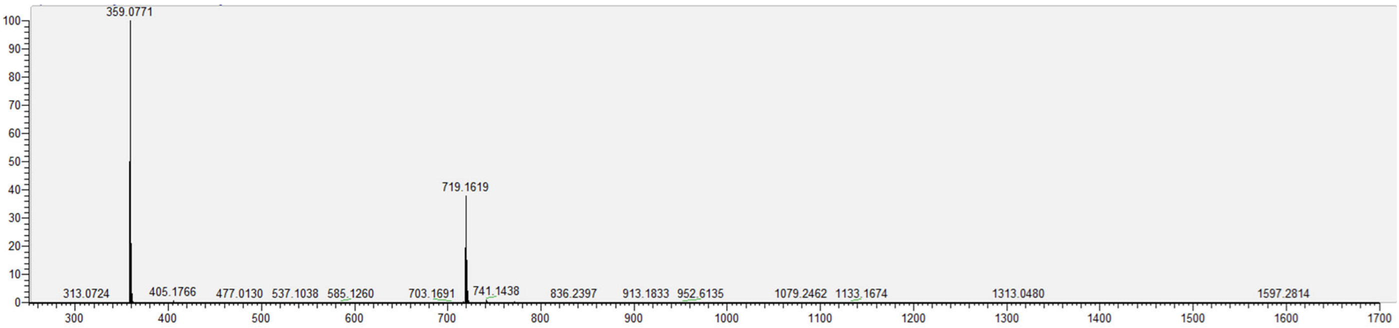

Figure S33. MS spectrum of Rosmarinic acid

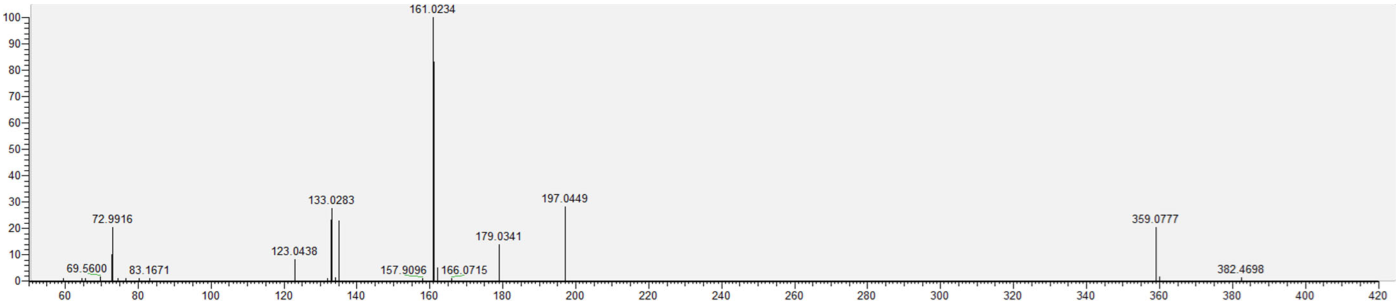

Figure S34. MS² spectrum of Rosmarinic acid

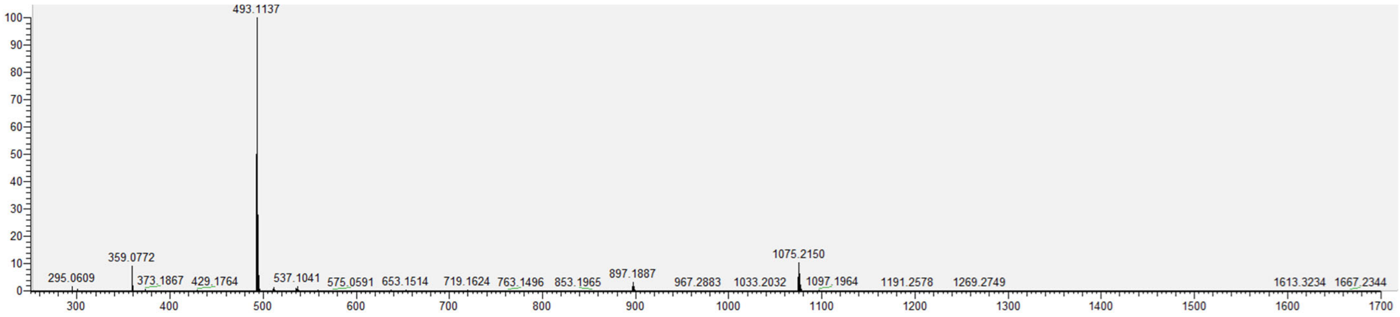

Figure S35. MS spectrum of Salvianolic acid A

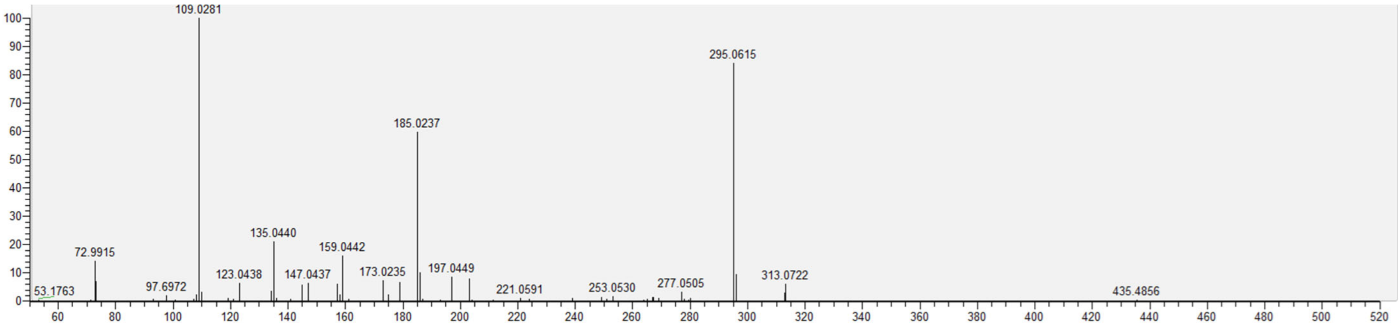

Figure S36. MS² spectrum of Salvianolic acid A

https://doi.org/10.3390/ijms27146122

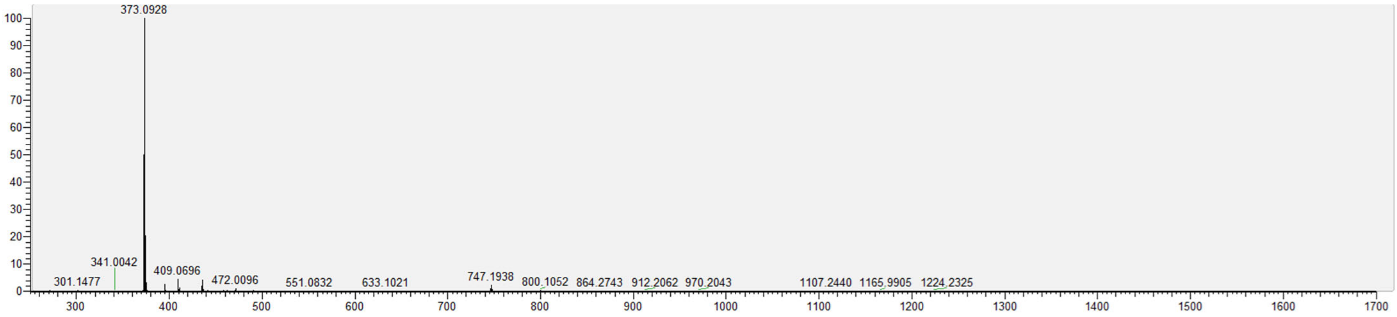

Figure S37. MS spectrum of Methyl rosmarinate

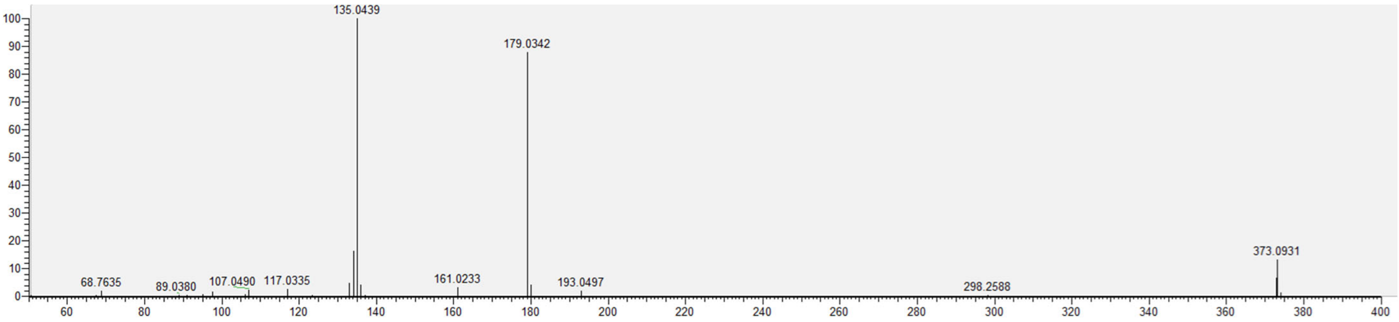

Figure S38. MS² spectrum of Methyl rosmarinate

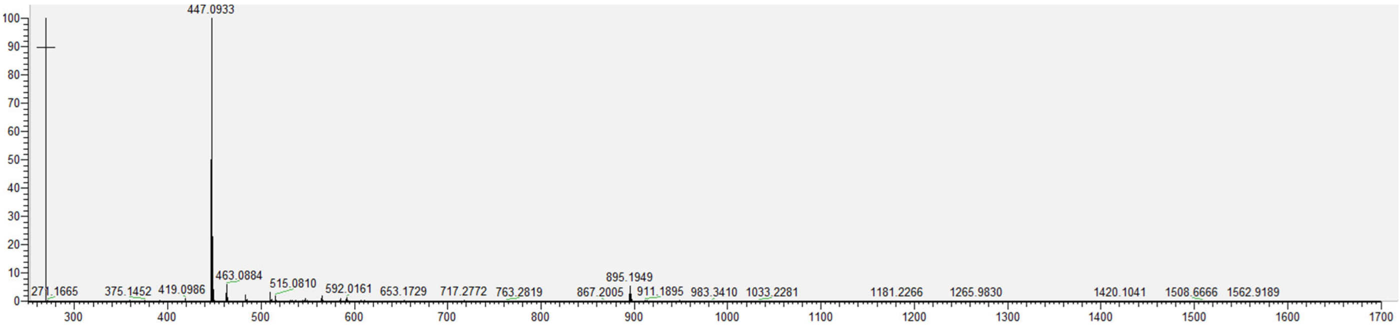

Figure S39. MS spectrum of Luteolin-hexoside

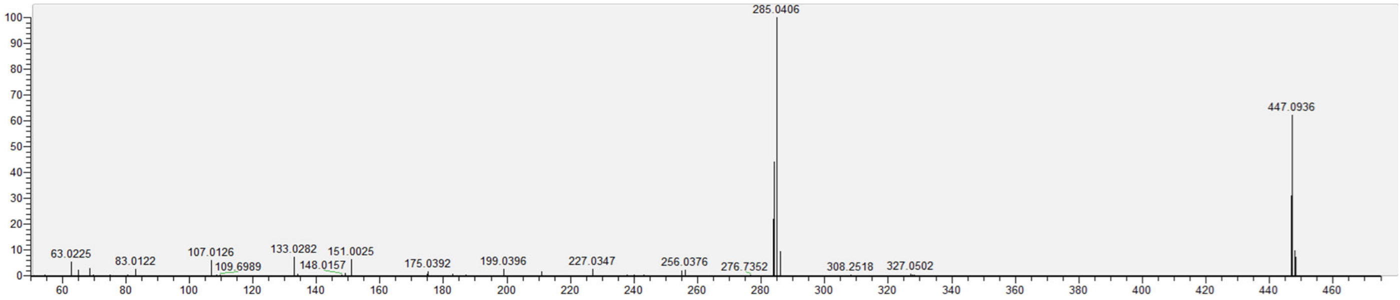

Figure S40. MS² spectrum of Luteolin-hexoside

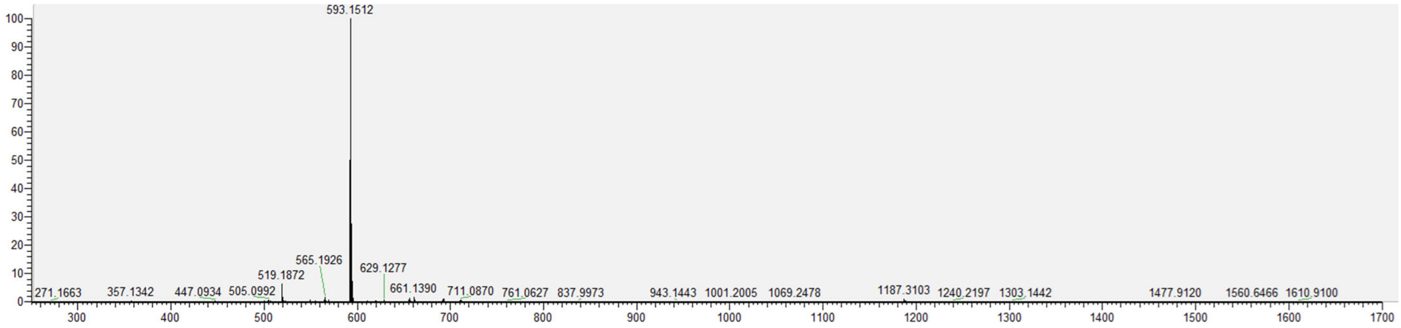

Figure S41. MS spectrum of Kaempferol-methylpentosyl-hexoside

https://doi.org/10.3390/ijms27146122

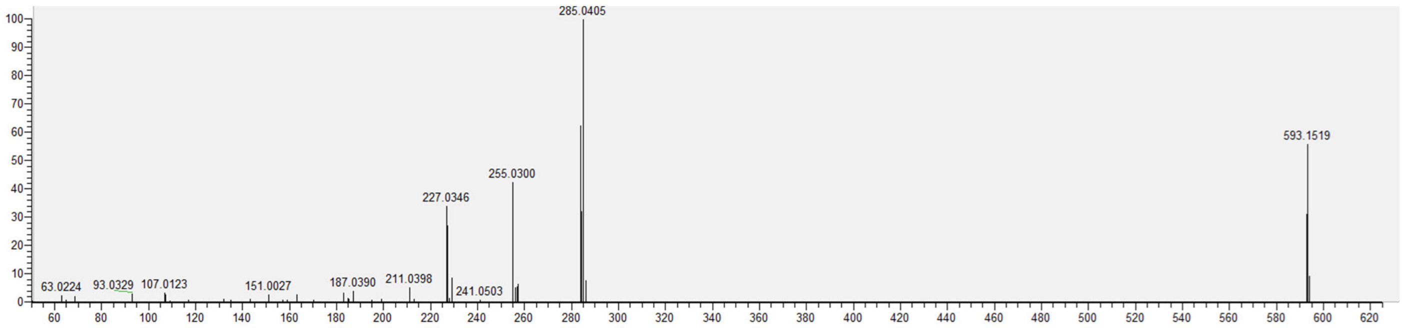

Figure S42. MS² spectrum of Kaempferol-methylpentosyl-hexoside

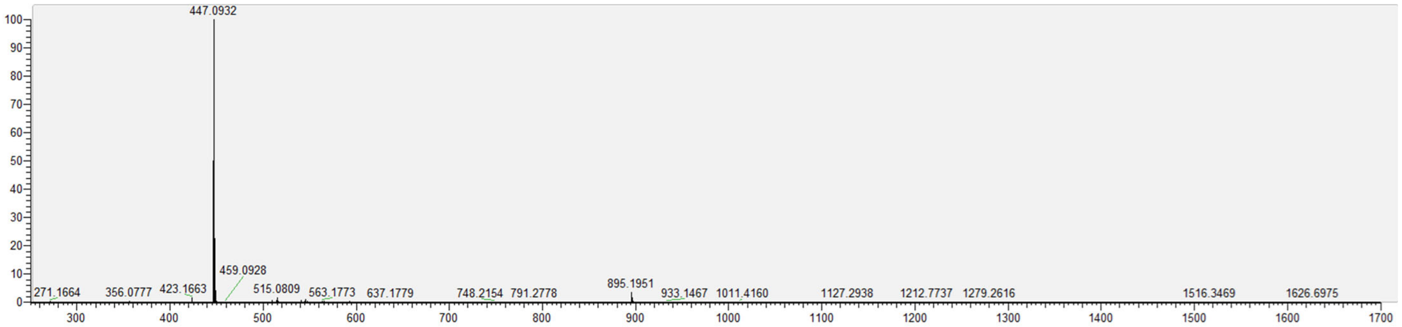

Figure S43. MS spectrum of Kaempferol-hexoside

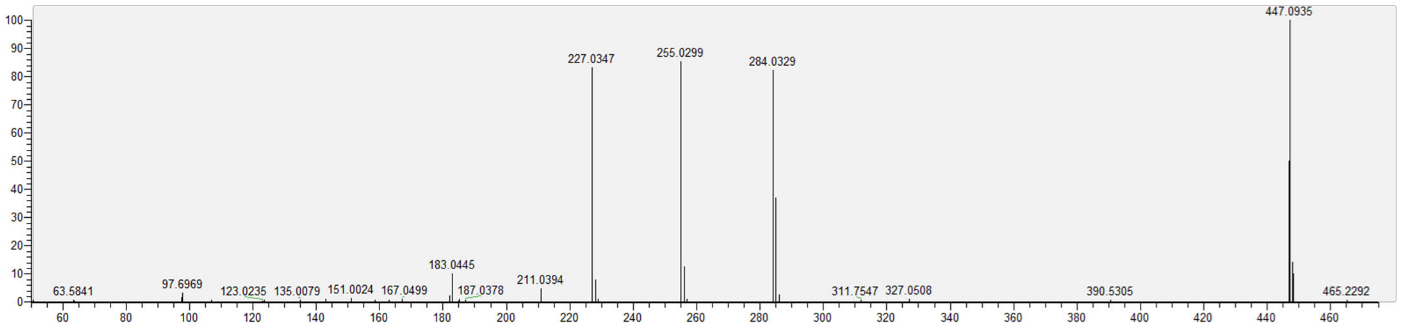

Figure S44. MS² spectrum of Kaempferol-hexoside

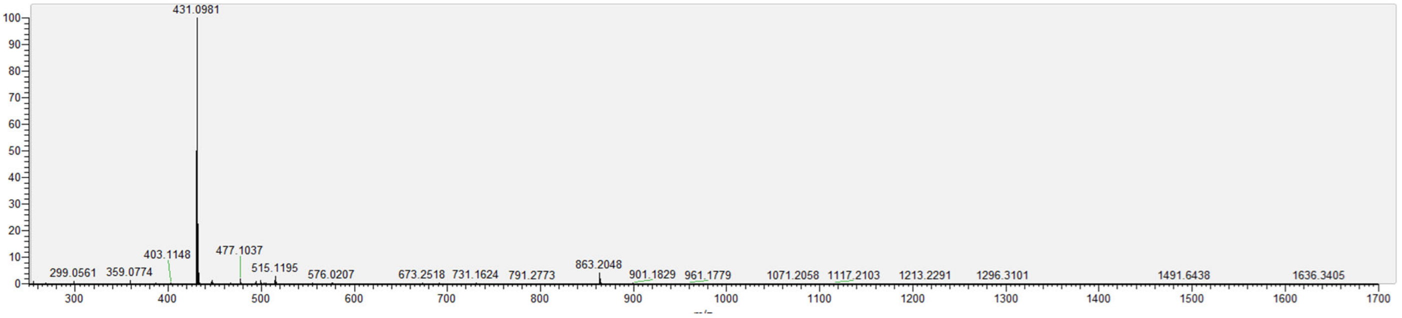

Figure S45. MS spectrum of Apigenin-hexoside

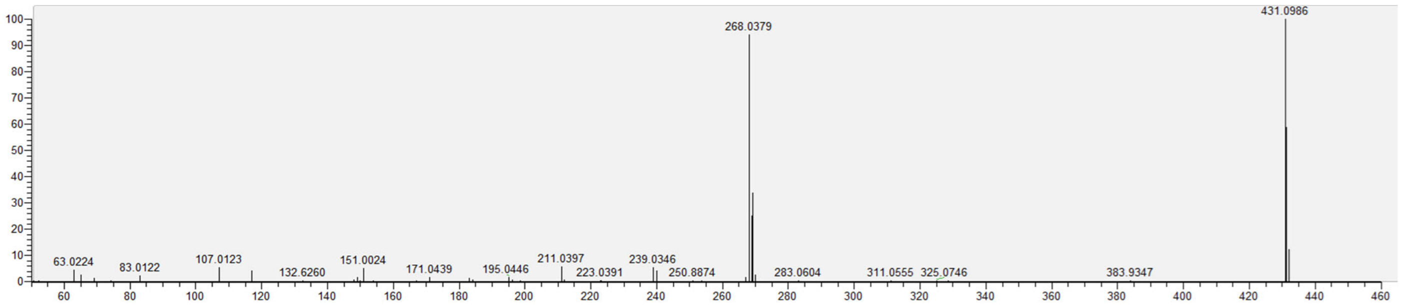

Figure S46. MS² spectrum of Apigenin-hexoside

https://doi.org/10.3390/ijms27146122

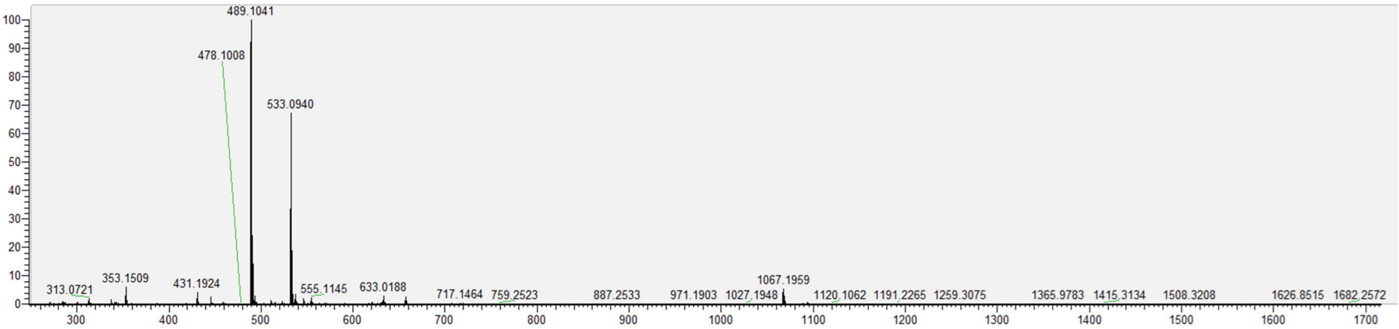

Figure S47. MS spectrum of Kaempferol-malonyl-hexoside

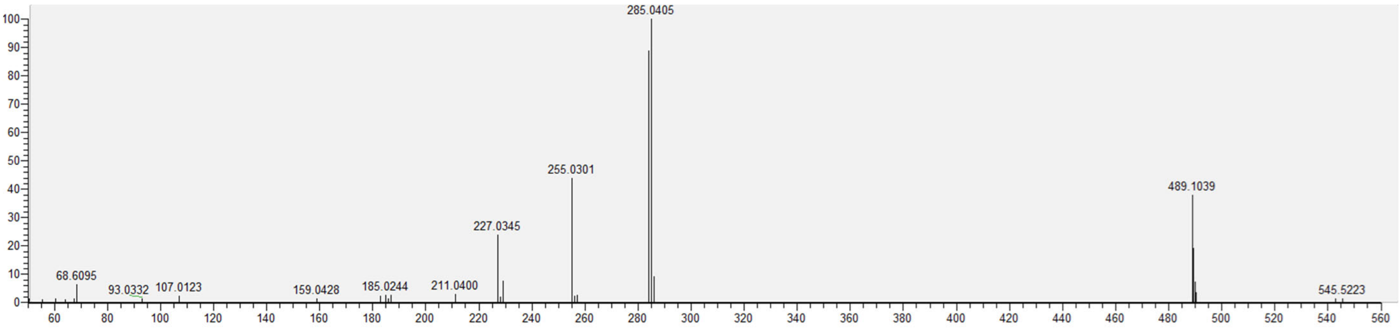

Figure S48. MS² spectrum of Kaempferol-malonyl-hexoside

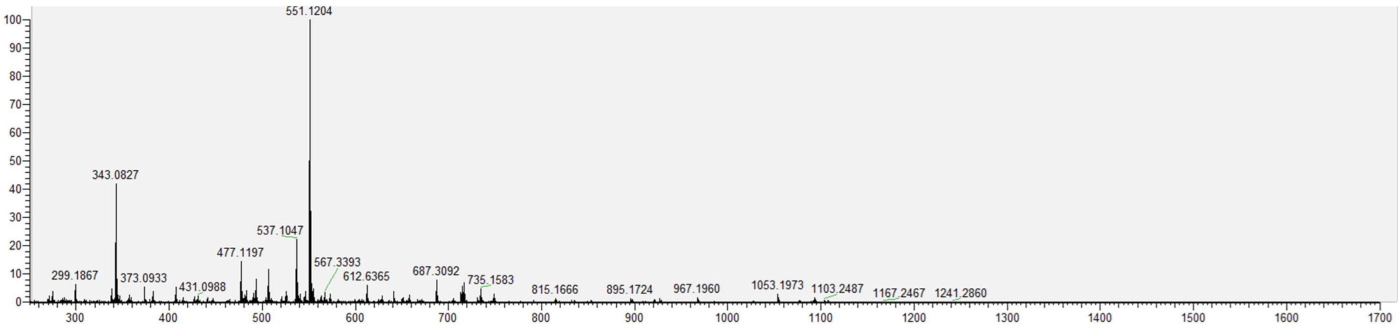

Figure S49. MS spectrum of Methyl ester of Salvianolic acid H

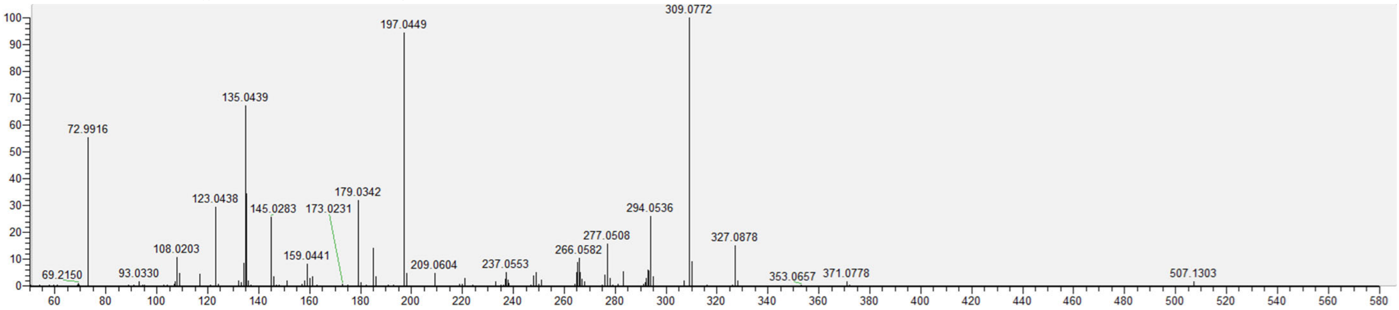

Figure S50. MS² spectrum of Methyl ester of Salvianolic acid H

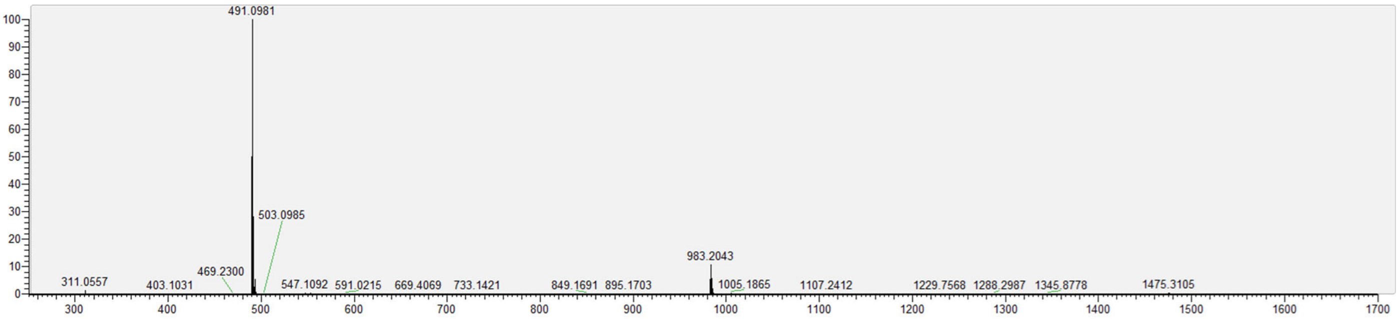

Figure S51. MS spectrum of Globoidnan A

https://doi.org/10.3390/ijms27146122

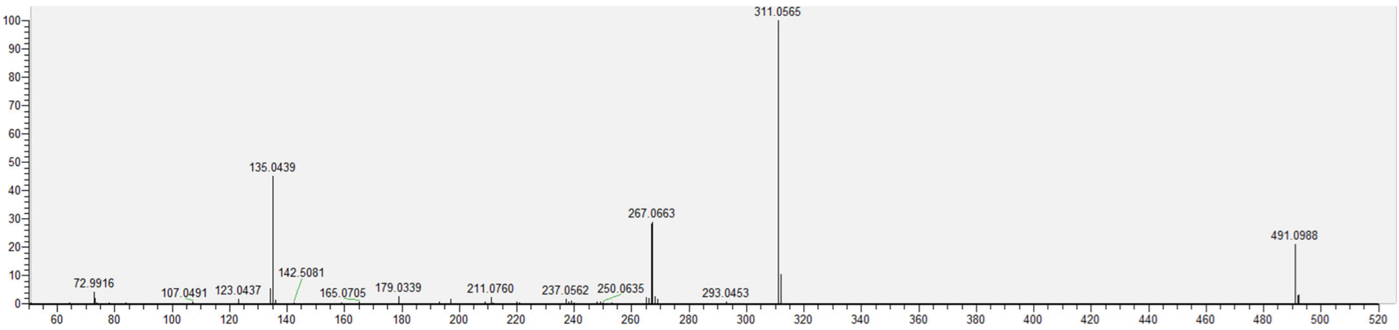

Figure S52. MS^2 spectrum of Globoidnan A

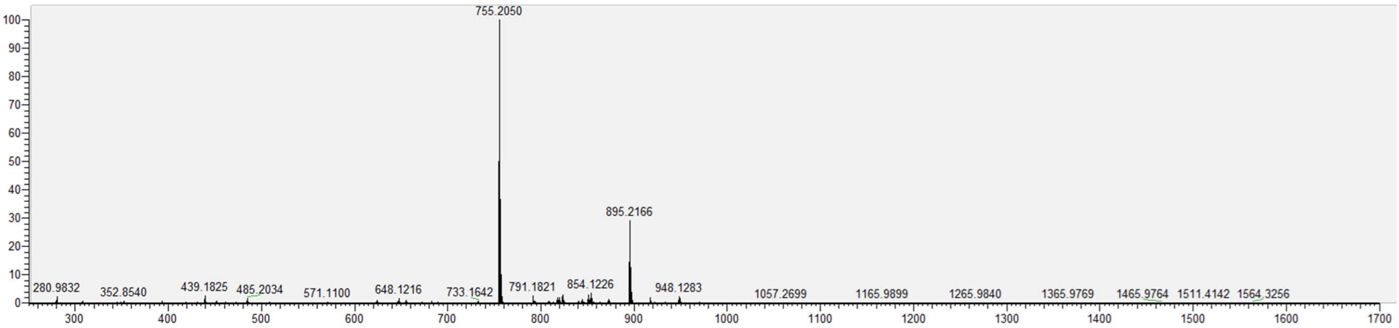

Figure S53. MS spectrum of Alcesefoliside

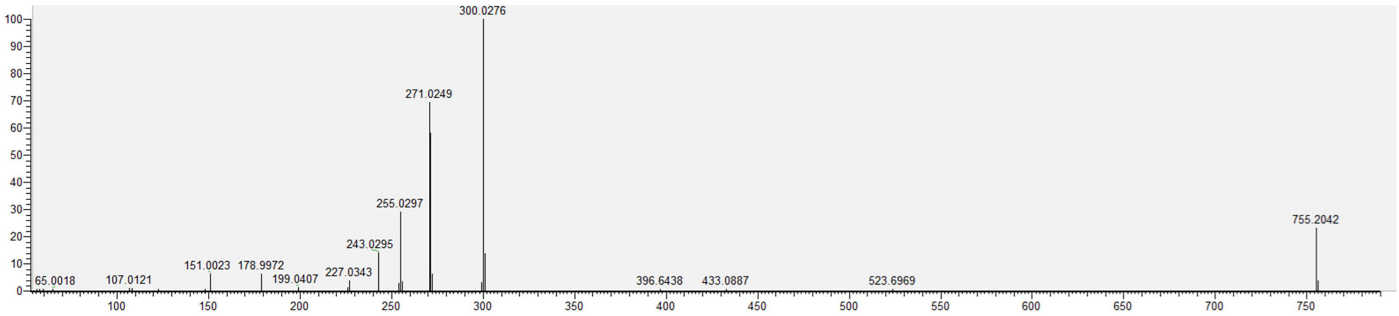

Figure S54. MS^2 spectrum of Alcesefoliside

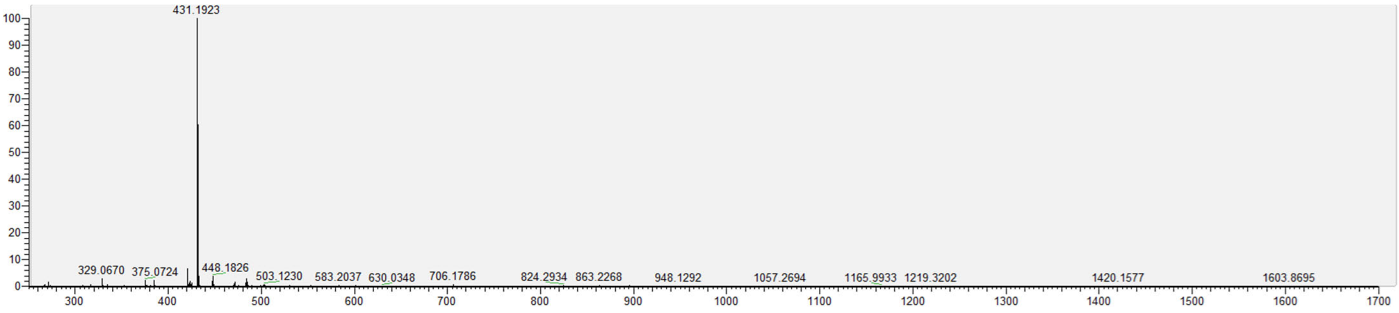

Figure S55. MS spectrum of Roseoside

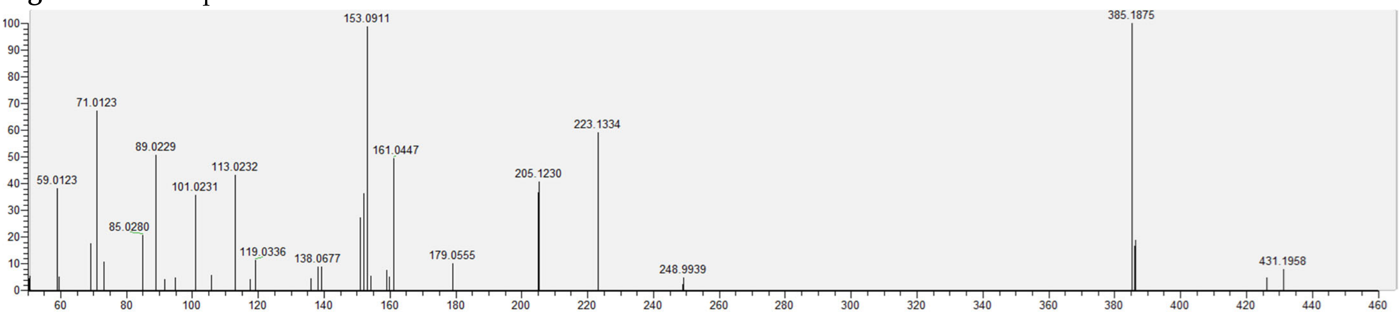

Figure S56. MS^2 spectrum of Roseoside

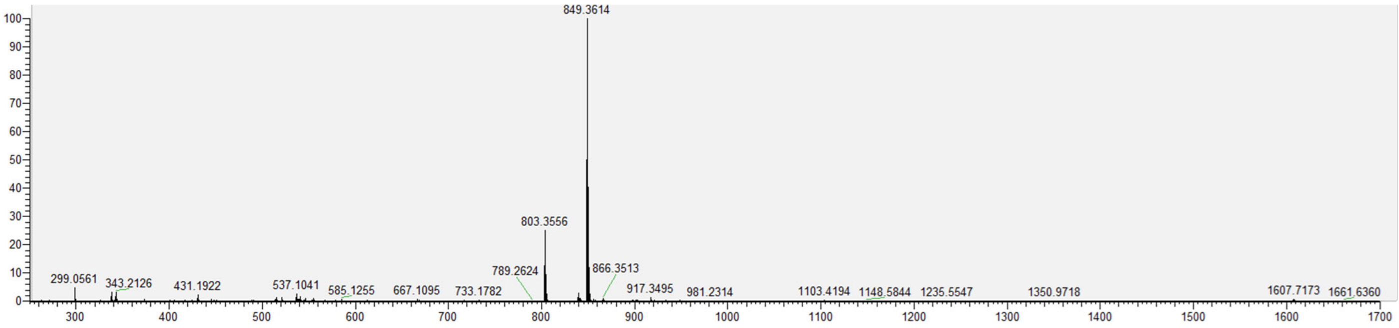

Figure S57. MS spectrum of Myricetin-trihexoside

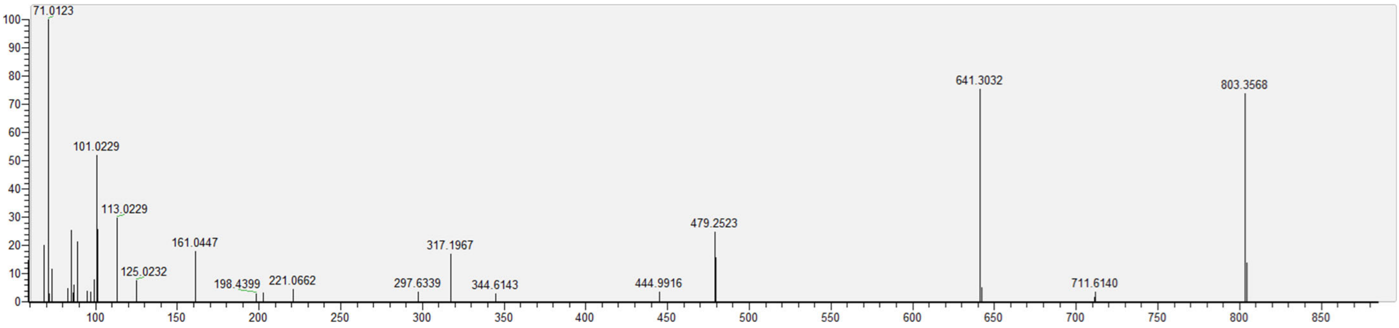

Figure S58. MS^2 spectrum of Myricetin-trihexoside

Disclaimer/Publisher's Note: The statements, opinions and data contained in all publications are solely those of the individual author(s) and contributor(s) and not of MDPI and/or the editor(s). MDPI and/or the editor(s) disclaim responsibility for any injury to people or property resulting from any ideas, methods, instructions or products referred to in the content.
